# Peptidomics Analysis of Soy Protein Hydrolysates—Antioxidant Properties and Mechanism of their Inhibition of the Oxidation of Palm Olein during Frying Cycles

**DOI:** 10.3390/foods12183498

**Published:** 2023-09-20

**Authors:** Annick Arcelle Pougoue Ngueukam, Mathilde Julie Klang, Ronice Zokou, Gires Teboukeu Boungo, Fabrice Djikeng Tonfack, Barakat Koyinsola Azeez, Hilaire Macaire Womeni, Apollinaire Tsopmo

**Affiliations:** 1 Biochemistry, Food Sciences, Nutrition and Medicinal Plants Research Unit, Department of Biochemistry, Faculty of Science, University of Dschang, Dschang P.O. Box 6, Cameroon; arcellepougoue@gmail.com (A.A.P.N.); klangjulie@gmail.com (M.J.K.); ronicezokou@gmail.com (R.Z.); giresteboukeu@yahoo.fr (G.T.B.); fdjikeng@gmail.com (F.D.T.); 2 Department of Biochemistry, Faculty of Science, University of Bamenda, Bambili P.O. Box 39, Cameroon; 3 School of Nutrition, Agriculture and Natural Resources, Catholic University Institute of Buea, Buea P.O. Box 563, Cameroon; 4 Food Science Program, Department of Chemistry, Institute of Biochemistry, Carleton University, 1125 Colonel by Drive, Ottawa, ON K1S 5B6, Canada; barakatazeez@cmail.carleton.ca

**Keywords:** antioxidants, protein hydrolysates, palm olein, oxidation, malondialdehyde, carbonyls, peptidomics, frying cycles

## Abstract

This study determined for the first time the structure of the peptides (i.e., peptidomics) in soy protein hydrolysates and elucidated their effects on an oil’s oxidative stability during frying cycles. The oil investigated was palm olein during 0, 4, 8, and 12 frying cycles of plantain banana chips. Proteins were extracted and hydrolyzed with two proteases. Trypsin hydrolysate (HTRY) exhibited higher anti-radical activity (DPPH, 70.2%) than the control (unhydrolyzed proteins, 33.49%) and pepsin hydrolysate (HPEP, 46.1%) at 200 µg/mL. HPEP however showed a 4.6-fold greater reduction of ferric ions (FRAP) while also possessing a higher peroxyl radical scavenging ability (716 ± 30 µM Trolox Eq/g) than HTRY (38.5 ± 35 µM Trolox Eq/g). During oil oxidative stability tests, HPEP improved the oxidative stability of the palm olein oil after 8 and 12 frying cycles, characterized by lower concentrations of hydroperoxides, and carbonyl and volatile compounds. HTRY however exerteda pro-oxidant activity. Structural data from SDS-PAGE and tandem mass spectrometry showed that the mechanism for the greater activity of the pepsin hydrolysate occurred due to unique structural features and a higher percentage of short-chain peptides. This was justified by a 25, 31, and 48% higher contents of tryptophan, histidine, and methionine, respectively (important amino acids with hydrogen atom transfer and electron-donating capacities) in the peptides identified in the pepsin hydrolysate.

## 1. Introduction

Worldwide, soybeans (*Glycine max*) and other oilseeds are mainly crushed to produce oilcakes and oils. Overall, the Food and Agriculture Organization (FAO) and the Organization for Economic Co-operation and Development (OECD) estimate that 90% of global soybean production will be crushed in 2027 compared to 86% for other oilseeds [1]. After the oil is extracted, the remaining solid residues can be transformed into a protein product called oilcake which is mostly used in the nutrition of cattle, sheep, pigs, poultry, bees, and shrimps [2]. Soybean meal is also considered a relatively cheap source of valuable phytochemicals like flavonoids due to their associated health benefits in a variety of formulated soy-based foods. The high protein content (ca. 62%) relative to the carbohydrate content (ca. 21%) of the oilcake contributes to its high nutritional value [3].

Edible oils resulting from crushing or other processes can undergo chemical alterations, including oxidation, during storage or cooking [4]. In recent decades, changing lifestyles (away from the workplace, out-of-home catering, unstructured eating, etc.) have contributed to the diversification of fried products, both in the catering and fast-food sectors and in the agri-food industry [5], as frying is a very common process used to prepare various food products with a pleasant taste [6]. During this cooking process, depending on the composition of the food to be fried, the type of oil used, and the frying conditions [7], several volatile and non-volatile compounds [8], many of which may be toxic, are formed during hydrolytic, thermal, and oxidative reactions.

The quality of oil after frying is affected by both the oil type and the nature of the food. Recent studies, for example, demonstrated that, during repetitive deep frying (French fries), sesame oil was more oxidized than palm oil due to the difference in their fatty acid composition and their degree of unsaturation [9], while a greater number of frying cycles was required to deteriorate, beyond acceptable quality, palm and canola (or rapeseed) oils than required to deteriorate corn and sunflower oils [10]. In another study, the deep frying of potatoes resulted in more oxidation of three types of oil (cold-pressed rapeseed, cold-pressed high-oleic rapeseed oil, and palm olein) than the deep frying of tofu [11]. High-carbohydrate foods such as potatoes and high-protein foods like tofu therefore have different effects on the quality of oils during deep-frying High-fat foods have also been reported to result in a greater oxidative degradation of oils (soy and mustard) during deep frying [12]. It is known that reactive oxygen species and free radicals resulting from oxidative reactions play a role in the onset of several diseases, such as atherosclerosis, high blood pressure, inflammation, cancer, diabetes, and Alzheimer’s disease [13]. In addition, oxidation affects the quality of oils and food due to the formation of oxidized fatty acids (peroxide, hydroxynonenal, and malondialdehyde) and, subsequently, oxidized amino acids or proteins, thereby reducing the shelf life of the oil or food and contributing to mutagenicity or carcinogenicity upon consumption [14]. Additives such as synthetic antioxidants butylhydroxytoluene (BHT), butylhydroxyanisole (BHA), and sulfites are efficient and commonly used to reduce lipid oxidation, although, due to potential carcinogenic effects, BHT and BHA are banned in some countries like Japan, Sweden, and Australia [15,16].

Studies have shown that peptides released from food proteins are multifunctional with antioxidant properties and have the ability to prevent the oxidation of lipids in model systems, such as linoleic acid emulsion and similar systems [17,18]. The application of hydrolyzed food proteins to assess the quality of fried oils is however limited. In the literature, protein hydrolysates were used to demonstrate a decrease in fat intake in fried foods, but there was no information provided on the quality of the fried foods [19,20]. A protein hydrolysate from sheep viscera (250–700 ppm) reduced the oxidation of soybean for 15 days at 63 °C, while the addition of amino acids in powder to soybean oil heated to 180 °C was very effective in preventing oxidation [21,22]. The effect of hydrolyzed proteins varies based on the type of oil, the food, the nature of the protease, and the profile of the peptides in the hydrolysates. The nature of the protein and the hydrolysis conditions will affect the balance of the hydrophobic versus hydrophilic residues and the proton-donating capacity, which will influence the capacity of an hydrolysate to act as antioxidants and stabilize oils or oil-containing foods.

The effect of hydrolyzed proteins on the quality of palm olein and the fraction of palm oil commonly consumed in many regions have not been investigated. This work therefore aimed to assess the effect of two hydrolysates of soy proteins on the oxidative stability of palm olein after various cycles during the frying of plantain banana, a variety of banana, and to correlate the results with the antioxidant activity of the hydrolysates and their peptide profile.

## 2. Materials and Methods

### 2.1. Materials

Soybeans (*Glycine max*), variety TGX1835-10C, were purchased from IRAD (Agricultural Research Institute for Development, Yaoundé, Camerun) Dschang and transported to the Biochemistry, Medicinal Plants, Food Science and Nutrition Research Unit of the University of Dschang. The seeds were soaked (100 g/200 mL) for 2 h in water and manually peeled to reduce the content of the anti-nutrient compounds, which can affect protein extraction yields, the protein’s quality, and their functions. They were then dried in an oven for 48 h at 45 °C in a Venticell^®^ electric oven (MMM Medcenter Einrichtungen GmbH, Munich, Germany). The dried seeds were crushed in a blender and sieved using a 200-micron mesh sieve. The flour obtained was packed in plastic bags and stored in a desiccator for the subsequent production of soybean meal.

### 2.2. Preparation of Soybean Meal

The soybean was defatted according to the method described by Womeni et al. [7]. Indeed, 100 g of soybean flour was macerated in 250 mL of hexane for 48 h and then filtered using muslin cloth and Whatman No. 4 paper. The residues were further defatted under the same conditions. After delipidation, phenolic compounds were eliminated via the maceration of the soybean meal in methanol using the method described by Iqbal et al. [23]. Similarly, 100 g of flour was mixed in 400 mL of methanol, macerated for 48 h, and then filtered using muslin cloth and Whatman No. 4 paper. The residues were again macerated under the same conditions to ensure that the majority of phenolic compounds were extracted. After delipidation and depolyphenolization, the cake was dried in an oven for 48 h at 45 °C, then packaged in plastic bags, and stored in the desiccator for later use.

### 2.3. Extraction of Proteins from Soybean Meal and Their Hydrolysis

Protein extraction was performed via alkaline solubilization and isoelectric precipitation according to the method described by Gnanasambandam et al. [24], with modification. Indeed, 20 g of soybean meal was solubilized twice in 100 mL of water, and the pH was adjusted to 9 using 2 N NaOH. Then, the mixture was stirred for 30 min at room temperature and centrifuged for 10 min at 4000 rpm in a benchtop TG18-WS centrifuge model (Drawell Anlytical, Shanghai, China). The supernatant was recovered; the proteins were precipitated at pH values between 4.5 and 5 with HCL 2 N and then centrifuged again at 4000 rpm for 10 min. The pellet obtained was dried in the Venticell^®^ electric oven (MMM Medcenter Einrichtungen GmbH, Munich, Germany) at 40 °C for 48 h. The extracted proteins were packaged in Whatman No. 4 filter paper and stored in the desiccator for later use.

Enzymatic hydrolysis was performed according to the literature [25] with slight modification. Into each beaker, 0.8 g of protein was introduced and homogenized with 100 mL of distilled water. Then, 0.1 g of enzyme was introduced and homogenized; the pH was adjusted to 7.4 for trypsin and 2.0 for pepsin. The 2 solutions were incubated for 15 min in a water bath at 40 °C and 30 °C, respectively. Hydrolysis was stopped by cooling and adding hydrochloric acid (HCl 1 N) to reduce the pH to 2 for trypsin and by increasing the pH to 8 with NaOH 1 N for pepsin. The hydrolysates were dried in the Venticell^®^ electric oven at 40 °C for 48 h and kept in the desiccator for later use.

### 2.4. Determination of Protein Content and Gel Electrophoresis (SDS-PAGE)

The amounts of soluble proteins in the extracted proteins and their digests or hydrolysates were determined using a modified Lowry assay [18]. Proteins, hydrolysates, and standard BSA (bovine serum albumin) were dissolved in 0.5 sodium dodecyl sulfate (SDS) with sonication (FS30, 100 W, 42 kHz, Fisher Scientific Co., Nepean, ON, Canada) when necessary. Seven concentrations of bovine serum albumin (10–200 µg/mL) were used to construct a standard curve, while the samples were analyzed at 100 µg/mL. The assay reagent was made by mixing 50 mL of reagent A (2.0% Na_2_CO_3_, 0.4% NaOH, 0.16% sodium tartrate, 0.5% SDS) and 0.5 mL of reagent B (4% CuSO_4_). To microcentrifuge vials, 200 µL of sample or standard was added, followed by 600 µL of the assay reagent and 10 µL of 1:1 water-diluted Folin–Ciocalteu phenol reagent (2 M). The mixtures were immediately vortexed and allowed to stand at room temperature (22 °C) for 45 min. The absorbance was recorded on triplicate samples at 660 nm on a BioTek^®^ Epoch™ UV-Vis microplate reader (Fisher Scientific, Nepean, ON, Canada). The soluble protein contents were calculated using the standard curve. SDS-PAGE was performed according to the method described by Walters et al. [26]. Samples were prepared with a protein content of 1 mg/mL. The samples were dissolved in a reducing buffer (0.125 M Tris-HCl pH 6.8, 4% *w*/*v* SDS, 20% *v*/*v* glycerol, and 0.5% 2-mercaptoethanol). The samples (20 μL) were loaded onto a 4% stacking gel and run on a 12% acrylamide gel for close to an hour at 150 V using a PROTEAN^®^ Tetra system (Bio-Rad Laboratories Ltd., Mississauga, ON, Canada). The gels were run in a 1X running buffer (0.303% *w*/*v* Tris base, 1.44% *w*/*v* glycine, 0.1% *w*/*v* SDS).

### 2.5. Mass Spectrometry Analysis

The tandem mass spectrometry (LC-MS/MS) system included a Dionex UltiMate 3000 nano LC linked to an Orbitrap Fusion mass spectrometer (ThermoFisher, San Jose, CA, USA). Experiments were performed at the Quebec Genomics Center (Sainte-Foy, QC, Canada). The analysis was based on a previously described procedure [17,27]. Full-scan mass spectra (350 to 1800 *m*/*z*) were acquired in the Orbitrap using an AGC target value of 4e5 at a resolution of 120,000. MS/MS peak lists were analyzed using Mascot Version: 2.8.0 (Matrix Science, London, UK), which was set up to search the GlycineMax_8827_20220912_20220912 database assuming non-specific enzyme. Fragment and parent ion tolerances were 0.60 Da and 10.0 ppm, respectively. Scaffold (version Scaffold_5.2.2, Proteome Software Inc., Portland, OR, USA) was used to validate MS/MS-based peptide and protein identifications, with peptide and protein thresholds set to 95% or greater [28].

### 2.6. Evaluation of Antioxidant Properties

#### 2.6.1. Ability to Scavenge 2, 2-Diphenyl-1-picrylhydrazyl (DPPH•)

The antioxidant activity of different proteins and hydrolysates was evaluated using the 2,2-diphenyl-1-picrylhydrazyl radical, as described by Mensor et al. [29]. For this, 900 μL of methanolic solution of DPPH was added to 100 μL of the samples at different concentrations (12.5; 25; 50; 100; 200 μg/mL). Mixtures were kept at room temperature in the dark for 30 min, after which, the optical density was measured at 517 nm on a BIOMATE brand spectrophotometer (Thermo Scientific™ 840208400/EMD). The absorbance of all samples was measured against a blank. A synthetic antioxidant (vitamin C) prepared at the same concentrations as the sample (12.5; 25; 50; 100; 200 μg/mL) was used as a positive control. The radical scavenging activity was determined using the following formula:

$$Activity\;\left(\%\right)=\frac{OD\;of\;DPPH-OD\;of\;Sample}{OD\;of\;DPPH}\times 100$$

Here, *OD* = optical density; *DPPH* = 2,2-diphenyl-1-picrylhydrazyl.

#### 2.6.2. Evaluation of Ferric Reducing Antioxidant Power (FRAP)

The reducing power of the various protein hydrolysates was also evaluated by determining their ability to reduce iron (III) to iron (II), according to the method of Oyaizu [30]. In test tubes containing 100 μL of proteins or hydrolysates at different concentrations of 12.5, 25, 50, 100, and 200 μg/mL prepared in distilled water, 500 μL of a potassium phosphate-buffered saline solution (0.2 M, pH 6.6) and 500 μL of 1% aqueous potassium hexacyanoferrate [K3Fe (CN)6] were added. The whole solution was incubated for 30 min at 50 °C in a water bath, and 500 μL of 10% trichloroacetic acid was added. The mixture was allowed to stand for 10 min, and 500 µL of supernatant was removed and mixed with 500 µL of distilled water; this was followed by the addition of 100 μL of 0.1% FeCl_3_ aqueous solution. Butylhydroxytoluene (BHT) at a concentration of 2 mg/mL prepared under the same conditions was used to compare the reducing power of proteins or hydrolysate. A blank was made of all reagents, except for the proteins or hydrolysate. The optical density of the samples and that of the control were measured at 700 nm against this blank on the BIOMATE brand spectrophotometer (Thermo Scientific™ 840208400/EMD). An increase in the absorbance of the reaction mixture indicated an increase in the reducing power of the proteins or hydrolysate to reduce iron (III) to iron (II).

#### 2.6.3. Oxygen Radical Absorbance Capacity Assay

The proteins’ and hydrolysates’ (pepsin, trypsin) ability to quench peroxyl radicals ROO•) derived from AAPH was assayed according to the oxygen radical absorbance capacity (ORAC) procedure [18,31]. A potassium phosphate buffer (pH 7.4; 75 mM) was used to make samples (0.1 mg/mL), fluorescein dye (0.08 µM), glutathione (positive control, 0.1 mg/mL), and Trolox standards (5–100 µM). In 96-well black microplates, 120 μL of fluorescein and 20 μL of either the phosphate buffer (blank), Trolox standard, or sample were added successively. The microplate was then incubated at 37 °C for 20 min, followed by the addition of 60 μL of AAPH (140 mM in buffer) to each well of the microplate. Fluorescent data were recorded (485/20 nm excitation and 528/20 nm emission) for 50 min with 1 min reading intervals on a BioTek^®^ Cytation™ 5 model (Fisher Scientific, Nepean, ON, Canada). The peroxyl radical scavenging activity (ORAC value) is expressed as µM Trolox Equivalent (TE)/g sample.

### 2.7. Palm Olein Stabilization and Frying of Plantain Banana Chips

Amounts (2 kg) of palm olein without additives were packaged in 4 opaques vials. Then, 2 g of trypsin hydrolysate and 2 g of pepsin hydrolysate, previously solubilized in 5 mL ethanol (95 °C), were introduced into 2 vials of the palm olein without additives (1000 ppm); 0.4 g of butylhydroxytoluene (BHT) previously solubilized in ethanol (95 °C) was introduced into the third vial (positive control); and 5 mL of ethanol (95 °C) was introduced into the last vial (negative control). Oil samples were agitated 5 times a day and stored at room temperature (approx. 20 °C) in the dark for 3 days. One hundred grams of each oil sample was packaged in opaque vials and stored in the freezer for later analysis. The oil samples were introduced in turn into a Rowenta^®^ electronic fryer (Erbach, Germany) and heated to 180 °C. Approximately 100 g of plantain banana was introduced into the oil and fried for 5 min. After 4, 8, and 12 frying cycles (a cycle being 6 min) with the different oil samples, 100 g of each oil was taken, packed in opaque vials, and stored in a freezer after cooling for subsequent analysis.

### 2.8. Chemical Characterization of Frying Oils

Lipid quality was evaluated by determining the peroxide value (PV) using the IDF 74A:1991 standard spectrophotometer method [32], the anisidine value (AnV) was determined using the official AOCS Cd 18–90 “p-anisidine value” [33], and the total oxidation value (Totox) was determined using the method described by Cong et al. [34]. The thiobarbituric acid value (TBARS) was determined using the method described in the literature [19]. Briefly, to 1 g of the oil sample, 0.1% trichloroacetic acid (1 mL) was added and vortex-mixed, followed by the addition of thiobarbituric acid (1 mL, 0.375%) and 1 mL of hydrochloric acid (0.25 N). The content was stirred and heated in a water bath at 95 °C for 30 min. The tubes were cooled and centrifuged, followed by an absorbance reading of the aqueous phase at 532 nm. A blank consisting of all reagents, except for the oil, was included. The iodine value (IV) was determined using the AOCS Cd 1–25 official method [33], and the acid value (Av) was determined according to standard NFT60-204 of the French Association for Standardization [35].

### 2.9. Statistical Analysis

The results obtained are expressed as a mean ± standard deviation, and comparisons between dependent variables were made using an analysis of variance (ANOVA) at the 5% probability threshold. Student–Newman–Keuls Multiple Comparison Test was used for characterization data of protein isolates and their effect on the oxidative stability of the oils during the frying of the plantain banana chips. These analyses were performed using Graph Pad prism and Scaffold (version Scaffold 5.2.2, Proteome Software Inc., Portland, OR, USA).

## 3. Results

### 3.1. Soluble Protein Content of Soybean Protein Isolate, Hydrolysates, and Molecular Weights

**Figure 1:**
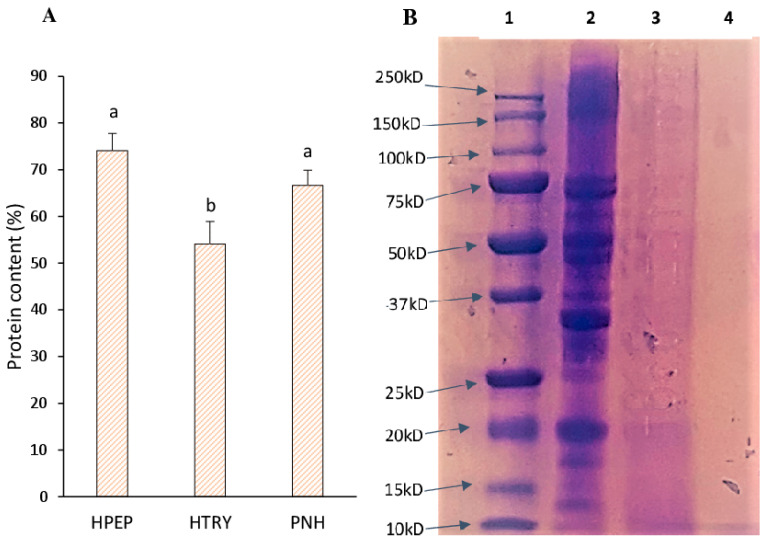
(**A**) Protein content and (**B**) SDS-PAGE of the soybean protein isolate and its hydrolysates. PNH: soy protein hydrolyzed with pepsin; HTRY: hydrolysate with trypsin; PNH: non-hydrolyzed soy protein. (1) Protein standard; (2) PNH; (3) HTRY; (4) HPEP. Data are means ± SEM (*n* = 3), and different letters indicate significant differences between means (*p* < 0.05).

The amounts of soluble proteins in the soy protein isolate (PNH) and its digests (HTRY and HPEP) are presented in Figure 1A. The pepsin hydrolysate and unhydrolyzed protein isolate had significantly elevated soluble protein levels (*p* < 0.05) compared to the trypsin hydrolysate. This result may be explained by the presence of non-protein compounds in the trypsin hydrolysate. The trypsin hydrolysate was also difficult to solubilize relative to the other two samples. The gel electrophoresis (SDS-PAGE) and mass spectrometry data confirmed the presence of larger polypeptides in the trypsin hydrolysate when compared to the pepsin hydrolysate. SDS-PAGE (Figure 1B) showed the main soy polypeptides conglycinins (α′, α, and β subunits) and glycinins (acidic and basic subunits) between 45 and 95 kDa and 20 and 37 kDa, respectively, in the isolate (PNH). In the trypsin hydrolysate, a small amount of the glycinin basic subunit (20 kDa) was present, while all polypeptides in the pepsin hydrolysate were less than 10 kDa. The profile of polypeptides in PNH is comparable to that in the literature data on soy protein isolates.

### 3.2. Mass Spectrometry Identification of Proteins and Peptides

The mass spectrometry data allowed for the identification of 75 proteins with molecular weights between 7 and 103 KDa in PNH (the non-hydrolyzed soy protein). In the meantime, the peptides in HTRY (trypsin hydrolysate) were derived from 57 proteins with molecular weights between 7 and 97 KDa, while those in HPEP (pepsin hydrolysate) originated from 38 proteins, with molecular weights between 15 and 97 KDa (Supplementary Table S1 and Supplementary Figure S1). The lower number of proteins indicated in the hydrolysates is due to hydrolysis in small fragments by each of the proteases. The data also showed that, even though PNH was not treated with any protease, it contained the largest number of peptides at 870, relative to 602 for the trypsin hydrolysate and 330 for the pepsin hydrolysate (Supplementary Figure S2). The decreased numbers of peptides in the hydrolysates indicate the actions of proteases, with pepsin having the greatest proteolysis action. The lengths of the peptides in PNH and HTRY were generally longer than those in HPEP. In the case of glycinin G4 (P02858), for example, the larger peptide sequences had 43 residues in PNH, 42 in HTRY, and 23 in HPEP, further illustrating the greater proteolytic action of pepsin. A complete list of the peptides in each sample is provided in Supplementary Table S2.

**Figure 2:**
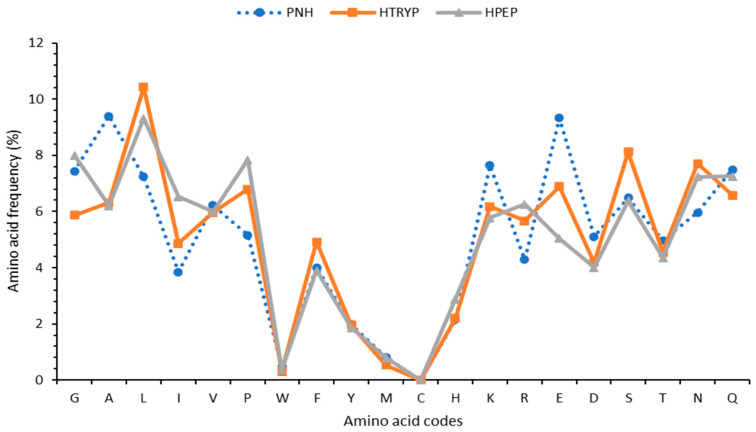
Amino acid frequency of different samples. HTRY: trypsin-hydrolyzed soy protein, HPEP: pepsin-hydrolyzed soybean protein, PNH: non-hydrolyzed soy protein.

Specific amino acids in peptides may contribute to their antioxidant properties; however, their overall sequences and the model system are likely more important [17]. The frequencies of tryptophan (Trp, W), histidine (His, H), and methionine (Met, M), some of the amino acids with hydrogen atom transfer and electron-donating capacities, were 25, 31, and 48% (Figure 2) in the total peptides identified in HPEP relative to those in HTRY, and this can justify the higher activity of HPEP (*p* < 0.05) in the ORAC and FRAP tests but not in the DPPH experiment, as its activity was lower (*p* < 0,05). In addition to a high content of most aromatic amino acids, the peptides in HPEP also had relatively high amounts of hydrophobic glycine (G, 36%) and isoleucine (I, 34%), while those in HTRY had a relatively high content of polar (acidic, basic, and neutral) amino acids, which were glutamic (E, 27%), aspartic (D, 4%), serine (S, 21%), lysine (K, 7%), and asparagine (N, 6%) amino acids (Figure 2). The overall activity of peptides depends on their sequences, their electronic properties, and the degree of hydrophobicity or hydrophobicity; as such, future work will use bioinformatics to select peptides for further investigation.

### 3.3. Antioxidant Activities

#### 3.3.1. Ability to Scavenge 2,2-Diphenyl-1-picrylhydrazyl (DPPH•)

**Figure 3:**
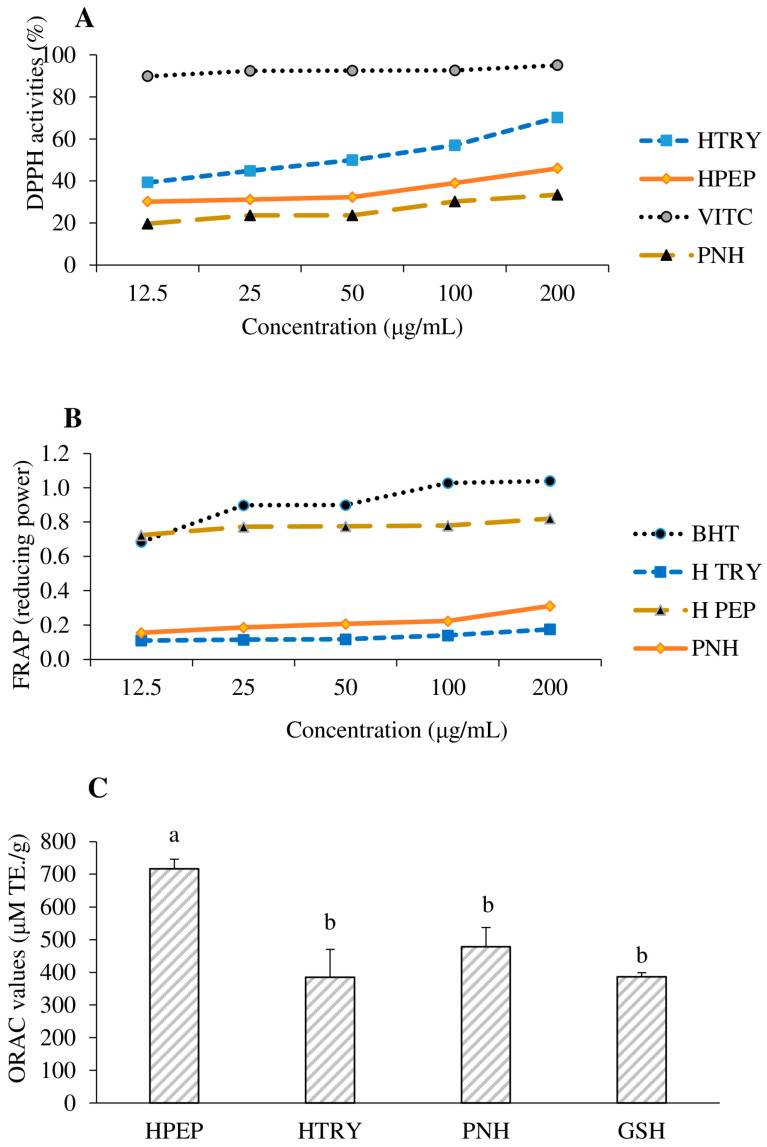
Antioxidant activities of samples. (**A**) DPPH radical scavenging activities at 12.5–200 μg/mL) with vitamin C (VITC as control); (**B**) FRAP (ferric reducing antioxidant power) with BHT (butylated hydroxy-toluene) as control; (**C**) ORAC (oxygen radical absorbance capacity) with GSH (glutathione) as control. HTRY: soy protein hydrolysate with trypsin, HPEP: soy protein hydrolysate with pepsin, PNH: non-hydrolyzed soy protein. Data are means ± SEM (*n* = 3), and different letters indicate significant differences between means (*p* < 0.05).

Figure 3A illustrates the anti-radical activity (DPPH scavenging) of the soybean protein isolate (PNH) and its hydrolysates (HTRY and HPEP) compared to that of a standard anti-radical agent, vitamin C. The soybean protein hydrolysates (HTRY and HPEP) showed significantly high DPPH anti-radical activity (*p* < 0.05) compared to the non-hydrolyzed soybean protein isolates. This result may be explained by the hydrolytic action of pepsin and trypsin, which may have released peptide sequences possessing proton-donating amino acids. Hamada as well as Fabian and Ju [36,37] showed that peptides with tyrosine and tryptophan at N-terminals exhibited the highest antioxidative activity. In another work, Bernardini et al. [38] showed that the aromatic amino acids tyrosine and histidine had stronger proton donor capabilities than other amino acids. Thus, the higher DPPH scavenging activity of HTRY at 200 µg/mL (70.19%) compared to that of HPEP and PNH (Figure 3A) could be related to its higher proportion of tyrosine- and histidine-containing peptides (51 sequences, Supplemental Table S2). Twenty-one had N-terminal tyrosine (Y), while one had that residue at both ends (YVVFKTHHNAVSSY) (see Supplementary Table S2). Babini et al. [39] showed that the presence of tyrosine and tryptophan in the C-terminal position of peptides is important for 2,2-azino-bis 3-ethylbenzothiazoline-6-sulfonic acid (ABTS) anti-radical activity. However, they also showed that, for 2,2-diphényl 1-pycrilhydrazyle (DPPH) anti-radical activity, the position of these amino acid residues is less important. This higher DPPH anti-radical activity of the trypsin hydrolysate could also be justified by the high arginine content of its MS-MS-identified peptides (see Supplementary Figure S1), compared to the non-hydrolyzed soybean protein isolate (PNH) and pepsin hydrolysate (HPEP). About 70 peptide sequences in HTRY have an arginine (R) residue at their C-terminal end. Indeed, arginine (R) residues, with their guanidine groups, would have the ability to complex or reduce DPPH radicals by giving them proton H^+^. Other basic amino acids, such as asparagine and glutamine, common in HTRY, can also, via their amide group, stabilize the DPPH radical using proton transfer. Additionally, the leucine (L) and proline (p) found in the identified peptides likely facilitate the hydrophobic interactions with free radicals rings, as suggested in the literature [40].

#### 3.3.2. Ferric Reducing Antioxidant Power (FRAP)

The reducing power of ferric ions is an antioxidant test used to assess the ability of a sample to prevent free radical formation by reducing iron (III) to iron (II) [30]. This test also makes it possible to evaluate the ability of a sample to inactivate certain types of reactive oxygen species responsible for lipid oxidation via electron transfer [41]. The reducing power of the hydrolyzed soybean protein hydrolysates (HTRY and HPEP) in comparison to that of BHT and the non-hydrolyzed soybean protein isolate is shown in Figure 3B. In general, it was noted that the reducing power of the different samples increased with the concentration. HPEP had a reducing power very close to that of standard butylhydroxytoluene (BHT) and significantly higher (*p* < 0.05) than that of the non-hydrolyzed protein and HTRY. The latter hydrolysate had a lower reducing power than the non-hydrolyzed protein. The high activity of the pepsin hydrolysate over the tested concentrations could be due to the high frequency of histidine (H) and its unique peptide sequences (Figure 2). Indeed, histidine has a good electron-donating capacity to convert ferric ions (Fe^3+^) into ferrous ions (Fe^2+^). Although the glutamic acid and aspartic acid within the peptide sequences may donate electrons and contribute to metal reduction [17], this does not appear to be the case in this work, as the HTRY peptides had a higher content of both acidic amino acids but a lower reducing power. Peptides with tryptophan at their C-terminal ends have been found to possess strong ferric ion reducing capacities [42], which might also explain the higher Fe^3+^ reducing capacity of HPEP. The shorter length of peptides in HPEP relative to that in the two other samples also explained the higher reducing power due to the better contact of proton-donating groups with the metal [17].

#### 3.3.3. Oxygen Radical Absorbance Capacity (ORAC) Properties

The peroxyl radical (ROO•) scavenging data of the soy protein isolate (PNH) and their hydrolysates (HPEP and HTRY) are shown in Figure 3C. They were obtained using the ORAC method, which measures the ability of compounds in a sample to scavenge ROO• radicals, mainly through proton-donating mechanisms. It was found that the pepsin hydrolysate (HPEP) had a higher scavenging capacity of 716 ± 30 µM TE/g (*p* < 0.05) than the controls (glutathione and PNH) and the trypsin hydrolysate (HTRY, 385 ± 86 µM TE/g). Pepsin had higher hydrolytic activity on the soy proteins, and this resulted in shorter peptide sequences; the mass spectrometry data showed a maximum of 29 residues for peptides in the pepsin hydrolysate compared to 42 residues for the peptides in the trypsin hydrolysate. This activity confirms that the low number of peptides observed in this hydrolysate was the consequence of the strong hydrolytic activity of this enzyme, which would have resulted in the release of short peptides with strong peroxyl radical scavenging and reducing activities. The higher antioxidant activity of HPEP may be due to increased exposure of a combination of internal hydrophobic and hydrogen atom-donating amino acids. In fact, the analysis of the mass spectrometry data showed that the contents of glycine, isoleucine, tryptophan, and histidine in the peptides present in HPEP were 25–36% compared to the contents of the same amino acids in the peptides present in HTRY.

### 3.4. Quality Chemical Indices

#### 3.4.1. Peroxide Value

**Figure 4:**
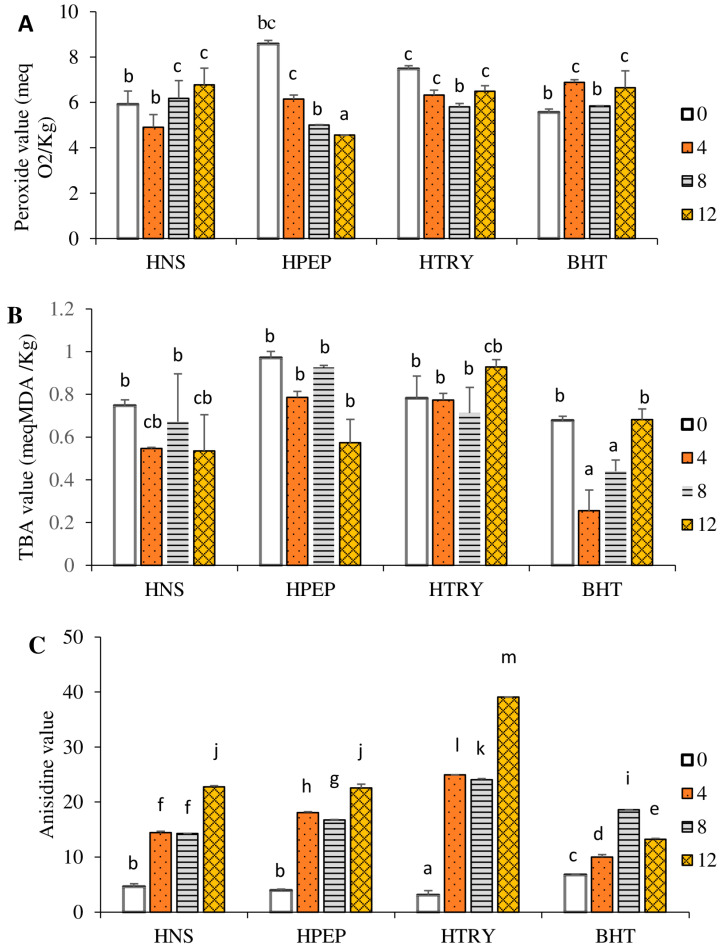
Oxidative indices of samples after 0, 4, 8, and 12 frying cycles. Cycle duration was 6 min. (**A**) Peroxide value; (**B**) thiobarbituric acid value; (**C**) anisidine value. HNS: unstabilized oil (no hydrolysate), HPEP: oil stabilized with pepsin hydrolysate, HTRY: oil stabilized with trypsin hydrolysate, BHT: oil stabilized with butylated hydroxytoluene. Data are means ± SEM (*n* = 3), and different letters indicate significant differences between means of all groups (*p* < 0.05).

The peroxide value is commonly used to determine the degree of primary oxidation products (mainly hydroperoxides) in an oil [34]. Thus, a high level of peroxide generally indicates an alteration in the oil, following the oxidation of its unsaturated fatty acids [43]. Figure 4A displays the peroxide values of the different oil samples depending on the number of frying cycles. Overall, the peroxide values of the control and experimental groups are within the standard reordered by the Stan Codex norm of less than or equal to 10 meqO_2_/kg of oil [44]. The peroxide value of the unstabilized oil (i.e., no hydrolysate) increased (*p* < 0.05) with the number of frying cycles. The initial addition of either hydrolysate led to a significant increase in hydroperoxides. Meanwhile, with an increasing number of cycles, the oil stabilized with HPEP showed a decreased level of oxidation (*p* < 0.05) from 8.6 ± 0.6 meq O_2_/Kg to 4.6 ± 0.2 meq O_2_/Kg. A lesser decrease in the level of oxidation was observed with HTRY, with no influence of the number of cycles from 4 to 12. The peroxide value of the oil stabilized with butylated hydroxytoluene increased significantly (*p* < 0.05) after 4 and 12 frying cycles compared to that of the negative control (T0). The increase in the peroxide value with the number of frying cycles of the unstabilized oil (no hydrolysate) is the consequence of an accumulation of hydroperoxides resulting from the oxidation of unsaturated fatty acids. These results are consistent with those of Debnath et al. [45], who showed that the peroxide value of rice bran oil increased with the number of cycles during deep frying. Similarly, the repeated frying of French fries (16 cycles, 5 min each) resulted in sesame oil having a greater peroxide value than palm oil, with the difference in oxidative level being attributed to the difference in the fatty acid compositions of both oils [9]. However, the decreased oxidation of the oils stabilized with the pepsin and trypsin hydrolysates with the number of frying cycles can be attributed to the presence of radical scavenging or reducing peptides in the hydrolysates, which limited the formation of hydroperoxides or transformed them into secondary oxidation products. High temperatures can convert hydroperoxides into aldehydes and ketones. It is therefore of interest to also quantify the secondary oxidation products in order to ensure the quality of the oils.

#### 3.4.2. Anisidine and Thiobarbituric Acid Values

The thiobarbituric acid (TBA) value is used to quantify the malondialdehyde (MDA) present or released in oil, while the anisidine value is used to quantify volatile secondary oxidation products such as 2-alkenal and 2,4-dienal. These compounds lead to the appearance of rancid odors in high-fat food products [46,47]. Figure 4B shows the TBA values of the different oil samples as a function of the number of frying cycles. Overall, the TBA values of the different oil samples are within the standard recommended by Codex Alimentarius [44], which is less than 2 meq MDA/kg oil. The TBA of the unstabilized oil decreased significantly (*p* < 0.05) after 4 cycles (T4) but not after 8 (T8) or 12 (T12) cycles, while no significant change was found with the addition of HTRY. The TBA value of the oil stabilized with the pepsin hydrolysate (HPEP) showed a significant decrease in MDA (*p* < 0.05) after 12 cycles compared to the other time points. A decrease in MDA concentrations (*p* < 0.05) was found with the control butylated hydroxytoluene after 4 and 8 cycles, with a subsequent increase to the initial value after 12 cycles.

The anisidine index (Figure 4C) of the different oil samples significantly increased (*p* < 0.05) with the number of frying cycles. The oils stabilized with the control BHT (butylated hydroxytoluene) showed an anisidine index (6.8–18.6) below 20 after all frying cycles, as recommended by the Codex Alimentarius [44]. The oil stabilized with the trypsin hydrolysate had the highest anisidine index (39.1 ± 0.2) after 12 frying cycles, while the values after 4 and 8 cycles were also about 25% above the recommended value. HPEP better prevented the formation of volatiles by maintaining anisidine values below 20 after 4 and 8 frying cycles and at 11.5% above the recommended value after 12 cycles. It can be concluded that, relative to HTRY, HPEP with a lower MDA concentration and anisidine index better limited the production of hydroperoxides and, consequently, their conversion into secondary oxidation products. The protective effect was however less than that provided by the control BHT. Additionally, the protective effect of HPEP was limited to 4 and 8 cycles, as the decrease in hydroperoxides after 12 cycles is associated with their greater conversion to rancid molecules (i.e., higher anisidine index). The stabilizing effect of HPEP correlates with its strong reducing and peroxyl radical quenching activities observed in the FRAP and ORAC data. This is likely due to it having a higher content of aromatic (e.g., histidine and tyrosine) and hydrophobic (e.g., isoleucine and glycine) amino acids than HTRY, which has more polar amino acids (Figure 2). The literature data indicate that a proper balance of polar and non-polar amino acids will facilitate the solubility of peptides in oil and, consequently, enhance their oil-stabilizing effects [48].

#### 3.4.3. Total Oxidation Value

**Figure 5:**
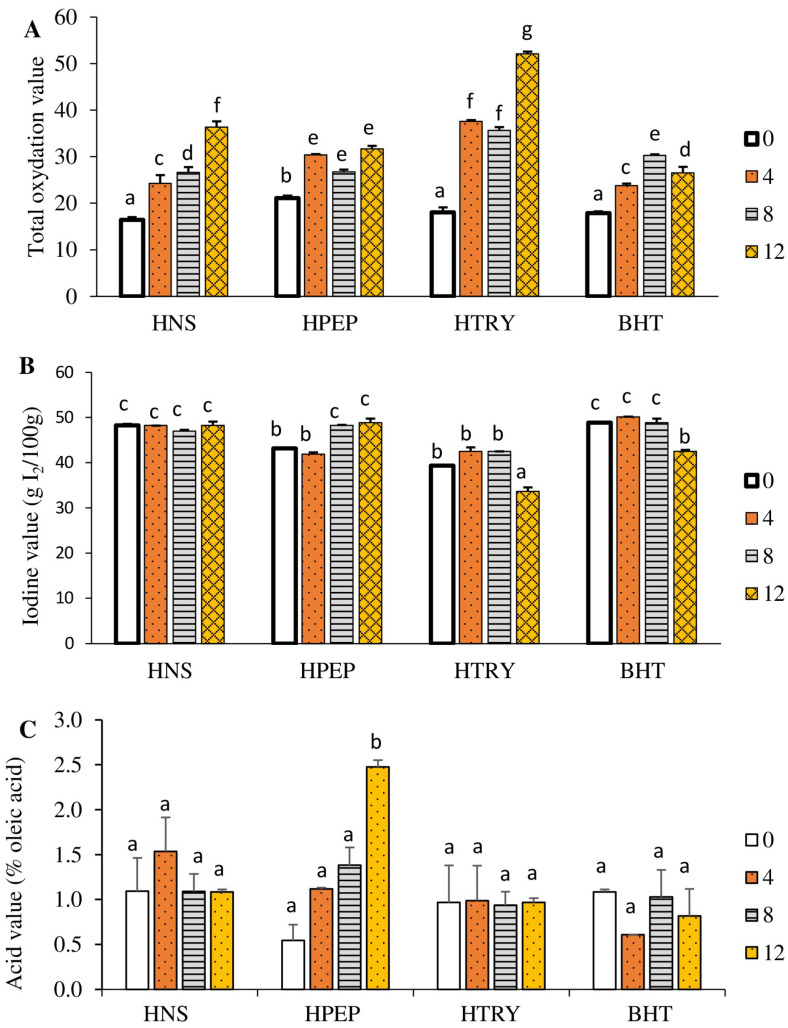
(**A**) Total oxidation index; (**B**) iodine values; and (**C**) acid values of samples after 0, 4, 8, and 12 frying cycles. Cycle duration was 6 min. HNS: unstabilized oil (no hydrolysate), HPEP: oil stabilized with pepsin hydrolysate, HTRY: oil stabilized with trypsin hydrolysate, BHT: oil stabilized with butylated hydroxytoluene. Data are means ± SEM (*n* = 3), and different letters indicate significant differences between means of all groups (*p* < 0.05).

Total oxidation (TOTOX) is a parameter that measures both primary and secondary oxidation products. It is obtained from the peroxide (PV) and anisidine (AN) values, using the formula TOTOX = 2PV + AN. According to the Codex Alimentarius [44], the TOTOX of an oil must be less than or equal to 26. Figure 5A shows that the total oxidation index of all oil samples increased significantly (*p* < 0.05) with the number of frying cycles, with one exception, the decrease from 30.4 to 26.8 between four and eight cycles. The highest value (52.1) recorded with the oil stabilized with the trypsin hydrolysate after 12 frying cycles demonstrated its pro-oxidant nature. The increase in the total oxidation value of all samples with the number of frying cycles confirms the notion that, at high temperatures, the oxidation of oils increases [9,10]. In contrast with trypsin, the oil stabilized with the pepsin hydrolysate had a relatively acceptable quality over the frying cycles with a TOTOX of 21.1–31.7. In general, the peptides identified in HTRY were larger, which likely decreased their solubility in the oil (palm olein). In systems such as emulsion, the peroxide and anisidine values were lower over 4 days in emulsions containing smaller peptides [49]. Other studies used protein hydrolysates in frying foods (e.g., fish and fish cakes), but they investigated the oxidative stability of the fried food and not that of the oil [19,20].

#### 3.4.4. Iodine Value

As for the iodine value, it is a parameter that measures the relative degree of unsaturation of fatty acids in an oil. The iodine values of the different oil samples as a function of the number of frying cycles are shown in Figure 5B. In this figure, it appears that the iodine value of the unstabilized oil remains constant with the number of frying cycles; however, the iodine value of the oil stabilized with the trypsin hydrolysate decreases significantly beyond eight frying cycles compared to that of the control (T0). In addition, the pepsin hydrolysate grows significantly (*p* < 0.05) beyond four frying cycles compared to the control; regarding the iodine value of the oil stabilized with butylated hydroxytoluene, the iodine value decreases significantly (*p* < 0.05) beyond eight frying cycles. The low iodine value of the oil stabilized with the trypsin hydrolysate after 12 frying cycles is explained by the massive destruction of the fatty acid double bonds of this oil at high temperatures, and it confirms the high value of the total oxidation value (52.1) previously recorded.

#### 3.4.5. Acid Value

Knowledge of the acid value allows for the quantification of free fatty acids in an oil; their presence is one of the factors causing alterations in an oil [50]. Figure 5C shows the acid numbers of the different oil samples as a function of the number of frying cycles. It appears that the acid number of all the oil samples remains significantly constant (*p* < 0.05) with the number of frying cycles compared to that of the control, except for the acid number of the oil stabilized with the pepsin hydrolysate, which increases significantly (*p* < 0.05) after 12 frying cycles. The high value of the free acidity, observed with the oil stabilized by the pepsin hydrolysate, may testify to the high hydrolysis of the ester bonds of the triglycerides of this oil under the effect of high temperatures. This result justifies the low peroxide value recorded with this oil sample, which is evidence of the effectiveness of pepsin hydrolysate in limiting the formation of primary and secondary products in frying oils. However, the low acid number values of the oil stabilized with the trypsin hydrolysate confirm its pro-oxidant effect at high temperatures. Although studies have shown increases in acid values with the number of frying cycles of oils, such as those from sesame, canola (also known as rapeseed), corn, and sunflower oils [9,10], no attempt has been made to stabilize them with additives.

## 4. Conclusions

The soy protein hydrolysate obtained from pepsin showed superior antioxidant activity over the one obtained from trypsin. Unstabilized palm olein showed a total oxidation product rate higher than the norm after eight frying cycles, while the oil stabilized with the trypsin hydrolysate showed a total oxidation product rate higher than the norm after all frying cycles. The oil stabilized with the pepsin hydrolysate showed a total oxidation product rate within the acceptable range up to eight frying cycles. The better performance of the pepsin hydrolysate was related to its greater proportions of smaller-sized peptides but, more importantly, to its higher content of protons and electron-donating amino acids, like tryptophan, histidine, and methionine, as well as the balance between hydrophobic and hydrophilic residues. Future works will attempt to improve the performance of the pepsin hydrolysate by optimizing the hydrolysis conditions.

## Data Availability

The dataset supporting the conclusions of this article is included within the article and Supplementary Materials.

## Supplementary Materials

The following supporting information can be downloaded at: https://www.mdpi.com/article/10.3390/foods12183498/s1, Figure S1: Venn diagram of the number of identified proteins in samples. PNH: non-hydrolyzed soy protein; HPEP: soy protein hydrolysate with pepsin; HTRY: soy protein hydrolysate with trypsin, Figure S2: Venn diagram of the number of identified proteins in samples. PNH: non-hydrolyzed soy protein; HPEP: soy protein hydrolysate with pepsin; HTRY: soy protein hydrolysate with trypsin, Table S1: Proteins identified in different samples and the number of peptides derived from each protein, Table S2: Peptide sequences of PHN, HTRY, and HPEP analyzed using LC-MS/MS and data validated by Scaffold software. PNH: non-hydrolyzed soy protein; HPEP: soy protein hydrolysate with pepsin; HTRY: soy protein hydrolysate with trypsin.
