# Supplementary material for: Peptidomics Analysis of Soy Protein Hydrolysates—Antioxidant Properties and Mechanism of their Inhibition of the Oxidation of Palm Olein during Frying Cycles
Source: Foods. 2023 Sep 20;12(18):3498. doi: 10.3390/foods12183498 (PMC10528889; doi:10.3390/foods12183498)
Supplement: Supplementary file 1 [file foods-12-03498-s001.zip › foods-2621164-supplementary.pdf]

Peptidomics analysis of soy protein hydrolysates-Antioxidant properties and mechanism of inhibition of the oxidation of palm olein during frying cycles

Annick Arcelle POUGOUE NGUEUKAM [1], Mathilde Julie KLANG [1], Ronice ZOKOU [1], Gires TEBOUKEU BOUNGO [1,3], Fabrice DJIKENG TONFACK [1,2], Barakat Koyinsola AZEEZ [4], Hilaire Macaire WOMENI [1,*], Apollinaire TSOPMO [4,*]

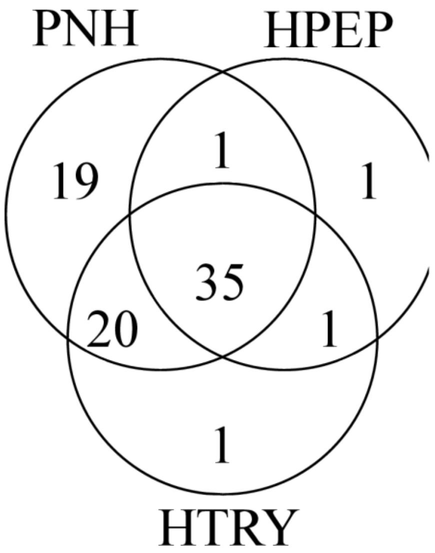

Supplemental Figure S1: Venn diagram of the number of identified proteins in samples. PNH: non-hydrolyzed soy protein; HPEP: soy protein hydrolysate with pepsin; HTRY: soy protein hydrolysate with trypsin.

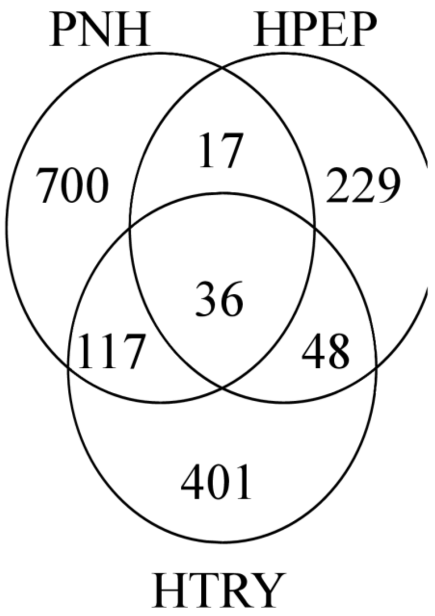

Supplemental Figure S2: Venn diagram of the number of identified peptides in samples. PNH: non-hydrolyzed soy protein; HPEP: soy protein hydrolysate with pepsin; HTRY: soy protein hydrolysate with trypsin.

Supplementary Table S1: Proteins identified in different samples and the number of peptides derived from each protein

Protein Name	Accession Number	Molecular Weight	Number of identified peptides		
			PNH	HPEP	HTPY
Beta-conglycinin alpha subunit 2	P0DO15 (+1)	70 kDa	81	55	98
Glycinin G4	P02858	64 kDa	113	34	84
Glycinin G2	P04405	54 kDa	105	34	102
Glycinin G1	P04776	56 kDa	30	25	51
Beta-conglycinin alpha' subunit	P11827	72 kDa	26	22	38
Uncharacterized protein (Fragment)	A0A0R0GMV1	55 kDa	28	17	27
Glycinin G5	P04347	58 kDa	6	3	7
Beta-conglycinin beta subunit 1	P25974	50 kDa	22	24	39
Glycinin G3	P11828	54 kDa	10	3	8
Basic 7S globulin	P13917	46 kDa	25	32	20
Basic 7S globulin 2	Q8RVH5	47 kDa	16	3	11
Uncharacterized protein	I1L860	58 kDa	18	19	18
Seed biotin-containing protein SBP65	Q39846	68 kDa	38	0	5
Uncharacterized protein	I1JF86	58 kDa	4	5	3
Lectin	P05046	31 kDa	13	7	13
Uncharacterized protein	A0A0R0JWF4	85 kDa	34	0	3
Uncharacterized protein	A0A0R0HYM3	70 kDa	7	3	5
Lipoxygenase	B3TDK6	97 kDa	11	5	8
Uncharacterized protein	I1L957	49 kDa	31	0	1
Seed maturation protein	Q9XET1	18 kDa	19	1	5
Trypsin inhibitor A	P01070 (+1)	24 kDa	4	7	8
Maturation protein	Q42447	26 kDa	14	0	3
Lipoxygenase	A0A0R0H569 (+1)	93 kDa	4	7	3
Late embryongenesis abundant protein	Q39871	51 kDa	3	0	0
Uncharacterized protein	I1L849	27 kDa	18	0	1
Uncharacterized protein	K7LEQ5	27 kDa	4	1	3
51 kDa seed maturation protein	Q39801	51 kDa	12	0	1
P24 oleosin isoform B	P29531	23 kDa	7	1	0
Lea protein	Q39873	49 kDa	8	0	0
Uncharacterized protein	I1JPV0 (+1)	35 kDa	10	0	0
34 kDa maturing seed protein	O64458 (+1)	43 kDa	5	5	4
Uncharacterized protein	I1NGG4	26 kDa	10	0	0
Pepsin A Cont_type=Reagent	P00791-CONT	41 kDa	0	8	0
Uncharacterized protein	I1LE41	26 kDa	2	0	0
Ribos_L4_asso_C domain-containing protein	I1L8R1 (+1)	45 kDa	11	1	0
Seed maturation protein PM30	Q9XET0	15 kDa	6	0	1
Maturation protein	Q01527	16 kDa	8	0	0
Bet_v_1 domain-containing protein	C6T588	17 kDa	5	0	0
Protein disulfide-isomerase	I1KAB7	56 kDa	4	1	0

Dehydrin	O23957	17 kDa	7	0	0
Formate dehydrogenase, mitochondrial	C6T9Z5	43 kDa	4	0	1
Cysteine proteinase inhibitor	I1M0K3	28 kDa	7	0	0
Cupin type-1 domain-containing protein	I1LHP6	82 kDa	2	0	1
60S ribosomal protein L36	C6TK30 (+1)	12 kDa	3	0	1
Uncharacterized protein	A0A0R0FZI9 (+1)	96 kDa	4	0	0
SHSP domain-containing protein	C6T1V2	18 kDa	1	1	2
Peroxiredoxin	A0A0R0K508 (+1)	20 kDa	2	1	1
Oleosin	C3VHQ8	17 kDa	2	0	2
Oleosin	K7KTR9	23 kDa	0	0	2
Uncharacterized protein	K7LDT9	103 kDa	3	0	0
18 kDa seed maturation protein	Q01417	18 kDa	2	0	0
Histone H2B	A0A0R0GW87 (+9)	15 kDa	5	1	1
Seed maturation protein PM22	Q9XER5	17 kDa	2	0	1
Uncharacterized protein	A0A0R0GZT5 (+1)	27 kDa	0	0	3
Ribosomal_L6e_N domain-containing protein	I1MJL6	26 kDa	4	0	0
40S ribosomal protein S30	C6TEP1 (+1)	7 kDa	5	0	0
Uncharacterized protein	C6SWW4	22 kDa	0	2	3
Late-embryogenesis abundant protein	Q9ZTZ2	15 kDa	1	0	2
Calreticulin	A0A762	48 kDa	1	0	3
Uncharacterized protein (Fragment)	A0A0R0HVK7	22 kDa	2	0	1
Ribosomal protein L19	A0A0R0HWC8 (+7)	24 kDa	5	0	0
Trypsin Cont_type=Reagent	P00761-CONT	24 kDa	0	0	3
KOW domain-containing protein	I1M2B6	17 kDa	3	0	1
Seed maturation protein PM41	Q9SWB2	8 kDa	3	0	0
Napin-type 2S albumin 1	Q9ZNZ4	18 kDa	2	0	0
Uncharacterized protein	I1NAI0	16 kDa	2	0	2
Uncharacterized protein	I1KZ37 (+1)	7 kDa	3	0	1
Uncharacterized protein	A0A0R0FEX5 (+3)	10 kDa	3	0	1
Uncharacterized protein	C6TFY7	11 kDa	2	0	0
EF1_GNE domain-containing protein	I1LWB8	24 kDa	3	0	0
Nucleoside diphosphate kinase	I1KJI7	17 kDa	0	2	0
Protein SLE1	I1N2Z5	12 kDa	2	0	0
Uncharacterized protein	I1LVC1	15 kDa	3	0	0
Uncharacterized protein	C6SYG6 (+1)	22 kDa	2	0	0
EF1_GNE domain-containing protein	I1M786	24 kDa	2	0	0
V-type proton ATPase subunit G	C6SV85 (+2)	12 kDa	2	0	0
Annexin	A0A0R4J4L3 (+1)	36 kDa	2	0	0
2S seed storage albumin protein	P19594	18 kDa	2	0	0

HTRY = soy protein hydrolyzed by trypsin, HPEP = soy protein hydrolyzed by pepsin, PNH = non-hydrolyzed soy protein

Supplementary Table S2: Peptide sequences of PHN, HTRY, and HPEP analyzed using LC-MS/MS and data validated by Scaffold software. PNH: non-hydrolyzed soy protein; HPEP: soy protein hydrolysate with pepsin; HTRY: soy protein hydrolysate with trypsin.

PNH	HTRY	HPEP
AAAARPLVT	ADFYNPKAGR	AAHAIATGDLVS
AAAARPLVTV	ADFYNPKAGRISTL	AALQTPKGKL
AAAARPLVTVQ	AGANSLLNALPEEVIQHTF	AALQTPKGKLEKL
AAAARPLVTVQA	AGRISTLNSLTL	AENNQRNFL
AAAARPLVTVQAL	AGRISTLNSLTLPALR	AENNQRNFLAGEKDNVVRQ
AAAARPLVTVQALD	AGRISTLNSLTLPALRQFGLSAQ	AGDTGRQHGNIGGPY
AAAARPLVTVQALDSD	AGVTVSKL	AGKDNIVSSL
AAAARPLVTVQALDSDM	AHSANLRQSQVSEL	AGLGHAPISL
AAAARPLVTVQALDSDMA	AIGINAENNQR	AIHATQGSTQQLGPL
AADQIAGQTFNDVGRF	AIHATQGSTQQL	AIHATQGSTQQLGPLVT
AADQIAGQTFNDVGRFDEEE	AISREQVRALAPTK	AIHATQGSTQQLGPLVTV
AAEKAKSAGGTTASYVGEK	AISREQVRALAPTKK	AIPVNKPGRFESF
AAGSTTAQYVGEK	ALKPDNRIESEGGF	AIQNKRLFL
AAKRAVGYLSGK	AMLHIPVSTPGKFEEFFGPGGRD	AKSSSRKTISSEDEPFNL
AAQAKDATVQTGKSAAKVASDLRDKA	ANSLLNALPEEVIQHTF	AKSSSRKTISSEDKPFNL
AAQAKDATVQTGKSAAKVASDLRDKALATGWSAAH	ANSLLNALPEEVIQHTFNLK	ALNGRALVQ
AATNVVKGVAE	APDTKRLADGLAF	ALQTPKGKLEKL
AATNVVKGVAEYAGQK	AQGRINEATQGAY	ALQTPKGKLERL
ADAAKRAVGYLSGK	AQGRINEATQGTY	AMLHIPVSTPGKF
ADLPGVKKEDLK	ASNLPHASSNID	AMLHIPVSVSTPGKF
ADLPGVKKEDLKVQVEENK	ASTDKSPDPTPAKGPD	AQQLPKQAQVKS
AEAQLRDQHGNPVPLTDQYGNPVILT	ATISDQPRG	AQQLPKQAQVKSVAPF
AEAQLRDQHGNPVPLTDQYGNPVILTD	AVAAKSQSDNFEY	AQRIPAGTTVY
AEAQLRDQHGNPVPLTDQYGNPVILTDE	AVAARSQSDNFE	ARAHTFVSPRHFDSE
AEAQLRDQHGNPVPLTDQYGNPVILTDERGNPVQL	AVQAKDVAVESGKSAAGY	ARPVLGGSSTFPYPR
AEAQLRDQHGNPVPLTDQYGNPVILTDERGNPVQLT	DAQRIPAGTTY	ARQIKNNNPFKF
AEAQLRDQHGNPVPLTDQYGNPVILTDERGNPVQLTGVA	DEVHGLYSGGIKLPTDIISKISPLPVL	ARQLEENL
AEKAKSAGGTTASYVGEK	DEVHGLYSGGIKLPTDIISKISPLPVLKE	ARQVKNNNPFSF
AEKLQSPDDERKQIVTVE	DNRIESEGGF	ASHFGLQRQ
AENNQRNFLAGSQDNVISQIPSQVQE	DPIYSNKLGKL	ASNLPHASSNIDPLDL
AETVSFSWNK	DTAEKLRSPDDERKQIVTVEGGL	AVEKLLKNQRESYF
AETVSFSWNKF	DTSNFNNQLDQNPRV	AVVAGLPVGGILL
AETVSFSWNKFVPKQPNMILQGDA	DTSNFNNQLDQNPRVFY	DKGIGTIISSPYR
AETVSFSWNKFVPKQPNMILQGDAIVTSSGK	DTSNFNNQLDQTPRV	DLNFTPREF
AEVIDDFAKF	EARAHTFVSPRHFDSE	EARAHTFVSPR
AEVIDDFAKFN	EARAHTFVSPRHFDSEVVF	EEINKVLFGRE
AEVRATGSNVITPGGLAAMAQSA	EDKGAIVTVKGGL	EEINRVLFGEE
AFGINAENNQRNFLAGEK	EEFFGPGGRD	EEKGAIVTVKGGL
AFGINAENNQRNFLAGEKDNVVRQ	EEINKVLFGREEGQQQGEERLQE	EITPEKNPQLRD
AFKPIAPR	EEINKVLFGREEGQQQGEERLQESV	EKLLKNQRESY
AFKPIAPRDAA	EEQRQQEGVIVE	FAIGINAENNQR

AFNYPSEMVNGVFDRK	ETLFKNQYGR	FAIGINAENNQRNFL
AFPGSAKDIENLIK	EYVSFKTND	FEITPEKNPQL
AFPGSAQAVEK	EYVSFKTNDRPSIG	FETRVETEGGR
AFPGSAQAVEKLLK	FASYPTKE	FGGIRAAPTGN
AFPGSAQAVEKLLKNQR	FASYPTKEESET	FGSLRKNAM
AFTPLTVTPQGEYNVRVSS	FAVAAKSQSDNFE	FSREEGQQQGEQRL
AHAHVDWRETD	FEITPEKNPQLR	FVVPPGHPFVT
AHAHVDWRETDK	FEITPEKNPQLRDLD	FYLAGNPDIEHPET
AHIFRADLPGVKKEDLK	FETKGEKKKDLVGRER	GALLLPHFNSKA
AIEGKDSAVGKLGEL	FGSNRFETL	GRVLIVPQ
AIEGKDSAVGKLGELK	FILRPDATYSIL	GRVLIVPQN
AIENVPDLKSYLGAR	FKNQYGRIR	GSRSQKQQLQDSHQKIR
AIGINAENNQRNFLAGSQD	FLAPIDTKPQTHAG	GSRSQQQLQDSHQKIR
AIGINAENNQRNFLAGSQDNVISQIPSQVQE	FLAPIDTKPQTHAGYL	GSRSQQQLQDSHQKIRH
AIPSEVLAHSYNLRQSQ	FLEHAFSVDKQ	GVAIKDPSTPHGVRL
AIVTSSGKL	FLEHAFSVDKQIAK	GVMNGGMQPRA
AIVTVKGGLR	FLKNDGTLRPLAIE	GVMNGGMQPRAE
AKDYTLQAAEK	FLKYQQQQQGGSQ	GVMNGGMQPRAEITL
AKDYTLQAAEKAKSAGGTTASYVGEK	FLSSTEAQQSYLQGFSR	HFNEGDVL
AKEAKDKTMEKGGEYKDYAAEK	FLSSTEAQQSYLQGFSRNIL	HNIGQTSSPDIYNPQAGSV
AKEGKDATVNKMGEYKDYAAEK	FLSSTEAQQSYLQGFSRNILEASYDTKF	IAEGHPLSL
AKEGKDATVNKMGEYKDYTAEK	FLSSTQAQQSYLQGFSKNILEASYDTKF	IAEGYPLSIKF
AKEGKDTTLGKLGELK	FLVPPQESQKRA	IAEKLQSPDDERKQIVT
AKEGKDTTLGKLGELKDSAADAAKRAVGYLSGK	FLVPPQESQRR	IGENKVAIVGVF
AKETKDKTMEKAGEYTDYASQK	FQKLDKESL	IGINAENNQRNF
AKGRAAADKEKGTKFAPED	FQTLFKNQYGHVR	IHIPAGTPLY
AKNAKDTTVQKAGEYKDY	FRLPENAKMDQVK	IIVVQGKGAIG
AKNAKDTTVQKAGEYKDYTAEK	FSHNILETSF	IIVVQGKGAIGF
AKSAGGTTASYVGEK	FSWNVLQAALQTPKGKL	IIVVQGKGAIGFAF
AKTTQTANKAMETGKDGKEAAESWTEWAKEKL	FVDAQPQQKEEGN	IIYALNGRA
AKTTQTANKAMETGKEGQEAAESWTEWAKEKL	FVDAQPQQKEEGSKG	IKDPSTPHGVRL
ALEPDHRVE	FVLSGRAILT	IKGRAVLGL
ALPEEVIQHT	FVVAARSQSDNFE	IKPPTDEQQQRPQE
ALPEEVIQHTFNLKSQQA	FVVPPGHPFVTIASNKENLL	ILGDVFIR
ALQAFQKFGNKL	GAIVTVKGGL	ILGDVFIRQY
APEFLKEAF	GAIVTVKGGLSVIKPPTD	ILGDVFIRQYYT
AQSFNTNEDTAEK	GKHQQEEENEGGSILSG	IPVNKPGRF
AQVELRDEHGNPVELTDAYGNPVWLTDEK	GLHLPSYSPYPQM	IRHFNEGDV
AQVELRDEHGNPVELTDAYGNPVWLTDEKGNPVHLTA	GLSVIKPPTD	IRQYYTVF
ARDVKERAKDYGSYAQGRINE	GRVLIVPQNFVVA	IRSIKTTSWDL
ARETKQKAGEYTDYAAQK	GSILSGFAPEFLEHA	IRVLKKFTEKSKL
ASELAAMSVDTAKGLAAAAAGENAKE	GSNRFETLF	ISKISPLPVL
ASELAAMSVDTAKGLAAAAAGENAKEYTARK	GTPKPSSLGRALY	ISKKQIRELSKH
ASHRQSYEAGQTKGRTE	GTRRLVVSKNKPLVVQFQKLDKES	ISKKQIRELSKHAKSSSR
ASHRQSYEAGQTKGRTEE	GTRRLVVSKNKPLVVQFQKLDKESL	ISLLDTSNFNNQLDQTPR
ASHRQSYEAGQTKGRTEEKTNQTMGNIGEK	HAAEKLSANQSHY	ITAFGGIRAAPTGN

ASKKQEERAEAAAK	IETWNPNNKPFQ	ITLAIPVNKPG
ASNRGQGGIQQLL	IGNLAGANSLL	IVFKTHHNAVT
ASNRGQGGIQQLLA	IHATQGSTQQLGPLVTVPQFL	IVTVKGGLRV
ASRQQFKEDRAE	IIIAQGKGALGVAIPG	IVTVKGGLRVTA
ASRQQFKEDRAEAAAK	IIPLPVIKEL	KIIGATNPAQSEPGTIRGDF
ASYQKHYDDQGR	IIVVQGKG	KIIKLAIPVNKPGRYDDF
ASYQKHYDDQGRKVD	IKLSSPATLNSR	KIRHFNEGDVL
ASYQKHYDDQGRKVDEYGNVE	ILRPDATYSIL	KLQSPDDERKQIVT
ATAVGWAAAHFSAEK	IPQHHTNPTKPINL	KSQQARQVKNNNPF
ATENLRAGVVGR	ISKEQIRALSKRAK	KSQQARQVKNNNPFSF
ATLVVHNQTSSAQDAEA	ISKKQIREL	KTISSEDEPFNL
ATLVVHNQTSSAQDAEAL	ISKKQIRELSKHAK	KTISSEDKPFNL
AVGWAAAHFSAEK	ISLLDTSNFNNQLDQNPRV	KTNDRPSIGNL
AVGYLSGK	ISLLDTSNFNNQLDQNPRVF	KTNDRPSIGNLAGANS
AVQAKDVAVESGK	ISLLDTSNFNNQLDQNPRVFY	KTNDTPMIGTL
AVQAKDVAVESGKSAAGYAAK	ISLLDTSNFNNQLDQTPRVF	KTNDTPMIGTLAGAN
AVQAKDVAVESGKSAAGYAAKVAADLRD	ISLLDTSNFNNQLDQTPRVFY	KVAKVLITY
AVQAKDVAVESGKSAAGYAAKVAADLRDKA	ISSNNFGKF	LAFGINAENNQRNF
AVRGAAPGHANTLGRPGVNFGGTTVMGVEPLHEL	ISTLNSLTLPALR	LAFPGSAKD
AVRLVLPGEL	ISTSTPHMVLQQSL	LAGEKDNVVRQIE
AVRLVLPGELAKHAVSEGTKAVTKFTSS	ITLAIPVNK	LAGNPDIEHPETM
AVTFSDPHTEEGIKSLDQFLSGK	ITLAIPVNKPGR	LAGSKDNVISQIPSQ
AVTFSNLHTESGLKSLDD	ITLAIPVNKPGRFE	LAIPVNKPGR
AVTFSNLHTESGLKSLDDFLSGK	ITLAIPVNKPGRFES	LAIPVNKPGRY
DAIVTSSGKL	ITLAIPVNKPGRFESFF	LAIPVNKPGRYDDF
DDDILGTLHD	IVEISKEQIRAL	LEHAFSVDKQ
DEDEDEDEDDEDEQIPSHPPRRPSH	IVEISKEQIRALSKRAK	LGPLVTVPQFL
DEDKQDLGVTGTG	IVEISKKQIR	LHIPVSTPGKF
DEEEPTWWER	IVFKTHHNAVTSYLKDVF	LKKQRESYF
DEMDEYPEVHGAPIYDSA	IVPQNFAVAAKSQSDNF	LKLAPGGHLGRF
DEVDEYPEVHGAPIYDSA	IVRNLQGENEEED	LKLSAQFGSL
DEVDEYPEVHGAPIYDSAPVR	IVRNLQGENEEEDSGA	LKLSAQYGSL
DEVHGLYSGGIKLPTD	KDVAVESGKSAAGY	LLLPHFNSKA
DGHQPGWQSVPTHDPQVQDAANHAIK	KEAFGVNM	LLLPHHADAD
DIAEKLQSPDDER	KETGSVASF	LLPHFNSKA
DIAEKLQSPDDERKQIV	KFLVPPQESQK	LLQKGLPRNIQG
DIAEKLQSPDDERKQIVTVE	KFLVPPQESQKRA	LNRNGLHLPSYSPYPQM
DIAEKLQSPDDERKQIVTVEGGLSVISPKWQEQQ	KGAIVTVKGGLSVIKPPTD	LQDSHQKIRHFNEGDVL
DIASEAGQYSAEK	KHQQEEENEGGSILSG	LRHNIGQTSSPDIYNPQAGSV
DIASEAGQYSTEK	KLHENIARPS	LRHNIGQTSSPDIYNPQAGSVTTA
DITAFGGIRAAPTGN	KLNALKPDNRIESEGGF	LRRPSYTNGPQE
DLAQKPVAPEDAAMMQSA	KNNNPFKFLVPPQESQKR	LRVPAGTTYY
DLPEGPAVKIGENKD	KNNNPFSFLVPPQESQRR	LVFTSPLHALF
DLRDKATAVGWAAAHFSAEK	KNQYGHVRVL	LVLSGRALINL
DMRTTPGGVAASMAAAATL	KNVRQIDVV	LVMDKPNGPVW
DMRTTPGGVAASMAAAATLNQN	KPDNRIESEGGF	LVMDKPNGPVWR

DMRTTPGGVAASMAAAATLNQNRQVTDA	KPLVVQFQKFRSST	LVMDKPNGPVWRISG
DNVAKELAFNYPSE	KPLVVQFQKLDKESL	LVNPDKKKDLRV
DQGRKVDEYGNVE	KRSPQLENLRD	LVNPHDHQNL
DQMPRRFYLAGNQEQEFLK	KSQQARQVKNNNPFSFLVPPQESQRR	LVQKGLPRNTQGV
DQNPRVFYLAGNPDIEHPE	KTISSEDKPF	MLGGLLHRGHKIKGTVVL
DQNPRVFYLAGNPDIEHPET	KTISSEDKPFNLRSRD	MLHIPVSTPGKF
DQNPRVFYLAGNPDIEHPETMQQ	KTNDRPSIGNLA	MNEGALLLPHF
DQNPRVFYLAGNPDIEHPETMQQQ	KTNDRPSIGNLAGANSLL	MQIVRNLQGEN
DQTPRVFYLAGNPD	KTNDRPSIGNLAGANSLLNALPEEVIQHTF	MRLRHNIGQTSSPDIYNPQAGSV
DQTPRVFYLAGNPDIEY	KTNDRPSIGNLAGANSLLNALPEEVIQHTFM[illegible]	MRLRHNIGQTSSPDIYNPQAGSVT
DQTPRVFYLAGNPDIEYPE	KTNDRPSIGNLAGANSLLNALPEEVIQQTF	NEGALLLPHF
DQTPRVFYLAGNPDIEYPET	KTNDTPMIGTLAG	NIQGVAGLGHAPISL
DQTPRVFYLAGNPDIEYPETM	KTNDTPMIGTLAGANSLL	NRNGLHLPSYSPYPRM
DQTPRVFYLAGNPDIEYPETMQ	KTNDTPMIGTLAGANSLLNAL	NSKPNTLLLPHHADA
DQTPRVFYLAGNPDIEYPETMQQ	KTNDTPMIGTLAGANSLLNALPEE	NSKPNTLLLPHHADAD
DQTPRVFYLAGNPDIEYPETMQQQ	KYQGNSGPLVNP	NSKPNTLLLPHHADADYL
DQTPRVFYLAGNPDIEYPETMQQQQQ	LAFPGSAQAVEKLLKNQRESYF	NSKPNTLLLPNHADA
DQTPRVFYLAGNPDIEYPETMQQQQQQ	LAFPGSAQDVERLLKK	NSLTLPALRQ
DQTPRVFYLAGNPDIEYPETMQQQQQQK	LAFPGSAQDVERLLKKQRESY	NVRVNSIRINQ
DQTPRVFYLAGNPDIEYPETMQQQQQQKS	LAGNPDIEHPE	PALRQFGLSAQ
DQTPRVFYLAGNPDIEYPETMQQQQQQKSHGGR	LAGNPDIEHPET	PGSAKDIENL
DQTPRVFYLAGNPDIEYPETMQQQQQQKSHGGRKQ	LAHSYNLRQSQ	PPNPHIGINVNS
DRHFKGEDFPEQNY	LAHSYNLRQSQVSE	PSISASGATPVF
DRHFKGEDFPEQNYIVKEGQLASQYR	LAHSYNLRQSQVSEL	PSYSPYPRM
DRPSIGNLAGANSL	LAIPVNKPGRFE	QEGRVLIVPQN
DSAADAAKRAVGYLSGK	LAIPVNKPGRFES	QLKYQGNSGPLVNP
DSGAIVTVKGGL	LDTSNFNNQLDQNPRV	QRIPAGTTYY
DSGAIVTVKGGLR	LDTSNFNNQLDQNPRVFY	RADLPGVKKEDL
DSVLKAELER	LDTSNFNNQLDQTPRV	RAHTFVSPR
DTAEKLRSPDDER	LEHAFSVDKQIAK	RAKSSSRKTISSEDEPFNL
DTAEKLRSPDDERKQ	LEHAFSVDKQIAKNLQ	RGSRSQKQQLQDSHQKIR
DTIGSVDHITTPLAEK	LEHAFSVDKQIAKNLQGE	RGSRSQQQLQDSHQKIRH
DTISSAAKTASEKTAPVAEK	LEHAFSVDKQIAKNLQGENEGED	RGSRSQQQLQDSHQKIRHFNEGDVL
DYASDATDAAKKTKDYAAQK	LEHAFSVDKQIAKNLQGENEGEDKGAIVTVKG	R[illegible]NKNPFL
EAVVGKKEPSTTTNSYSTR	LEHAFSVDKQIAKNLQGENEGEDKGAIVT[illegible]	[illegible]QF
EAVVGKKEPSTTTNSYSTRE	LFVLSGRAIL	RQFQNQDIF
EAVVGKPHHEEVYAKETKMGE	LHIPVSVSTPGKFEEFFGPGGRD	RQFQNQDIFHDLA
EEEGGSVLSGFSK	LHLPSYSPYPRM	RQIKNNNPFKF
EEEGGSVLSGFSKHF	LKLSAQYGSLRKNAM	RQNIGQNSSPDIYNPQA
EEEGGSVLSGFSKHFL	LKPDNRIESEGGF	RQNIGQNSSPDIYNPQAGSI
EEEGGSVLSGFSKHFLAQSFNTNEDIAEK	LKVREDENNPFYLR	RQNIGQNSSPDIYNPQAGSITT
EEHEWHRKEEKHGGKGSEEEQDE	LKVREDENNPFYLRSSNSF	RQNIGQNSSPDIYNPQAGSITTA
EEINRVLFGEEE	LLFVLSGRAIL	RRGSRSQKQQLQDSHQKIR
EEQDERQFPFPRPPH	LPEEVIQHTF	RSQQLQNLRD
EEVIQHTFNLKSQQAR	LQGFSKNILEASYDTKF	RTLNRNGLHL
EGSIFAISR	LQRFNKRSPQL	RVETEGGSIRVLK

EGSIFKISR	LQRFNKRSPQLENLRD	RVLIVPQN
EKAKSAGGTTASYVGEK	LRLITLAIPVNKPGRFESF	RVTAPAMRKP
EKGAIVTVKGGL	LRSRDPIYSNKLGKF	SAATGLDIPGESHDVL
EKLPADKAVTR	LSAEFGSLRK	SIDAKDFHL
EKLPADKAVTRE	LSSPATLNSR	SIIPNLFGGR
EKLPADKAVTREDAEGVIGAELRNKL	LSVIKPPTD	SKPNTLLLPNHADADYL
ELAFKPIAPR	LTLPALRQFGL	SREEGQQQGEQRLQESV
ELAFKPIAPRDAAL	LVLPVQNDASTGLHW	SRKTISSEDKPFNL
ELAFKPIAPRDAALMQA	LVLPVQNDASTGLHWANL	SRYPTSKGAIIF
ELAFNYPSEMVNGVFDRK	LVLPVQNDASTGLHWANLQKR	SVDKQIAKNLQGENEGEDKGAIVTVKGGL
ELAFPGSAQAVEK	LVLSGRALINLVKPDDRD	SVIKPPTDE
ELAFPGSAQAVEKLLKNQR	LVMDKPNGPVWRISGEDL	SVIKPPTDEQQQRPQEEEE
EPQQPGEKEEDEDEQPRPIP	LVPPQESQRR	SVISPKWQEQEDEDEDE
EQHQHDENKPGVIGSMFR	LVQKGLPR	SVISPPTEEQQQRPEE
EQIPSHPPR	LVQKGLPRN	SWWPFGGESKPQF
EQVRALAPTK	LVQKGLPRNTQG	TIIPLPVIKE
ERQQHGEKEEDEGEQPRPFP	LVVFDLAR	TIISSPYR
ESYFVDAQPKK	MIYDVNSPLFRSFL	TLAIPVNKPGRF
EVHGLYSGGIKLPTDIIS	MMSPQAASPGLVRAEL	TLVNNDDRDSYNLHPGDA
EVIQHTFNLKSQQAR	MQIVRNLQGENEEED	TNDRPSIGNL
FAIGINAENNQRNFLAG	MQIVRNLQGENEEEDSG	TVQVGGISDTNQIFGL
FAPEFLEHA	MQIVRNLQGENEEEDSGA	TVQVGGISDTNQIFGLS
FDLNFTPRE	MQIVRNLQGENEEEDSGAIVTVKG	VAGLGHAPISL
FGINAENNQRN	MVNGVFDRKESFF	VEISKEQIRALSK
FGINAENNQRNFLAGEK	NALEPDHRVES	VFRAIPSEV
FGINAENNQRNFLAGEKDNVVRQ	NALEPDHRVESE	VFVVPPGHPFVT
FGVNMQIVRNLQGE	NALEPDHRVESEG	VGIKEQQQKQKQEEEPLE
FKNQYGHVRVL	NALKPDNRIESEG	VIDGRGHL
FKNQYGHVRVLQ	NALKPDNRIESEGGF	VIKPPTDEQQQRPQE
FKNQYGHVRVLQR	NALPEEVIQHT	VLAIHATQGSTQQLGPLVT
FLAFGINAENNQRNFLAGEK	NGLHLPSYSPYPR	VLGGNDTFPYPR
FLAGEKDNVVRQ	NKNPFHFNSK	VLGGSSTFPYPR
FLAQSFNTNE	NKNPFHFNSKR	VMDKPNGPVWR
FLAQSFNTNEDIAEK	NKPLVVQFQKFRSST	VQKGLPRNTQGVAGLGHAPISLPNQL
FLAQSFNTNEDTAEK	NKRSPQLENLRD	VREDENNPFY
FLKYQQQQQGGSQSQKG	NLAGANSLL	VRVLQRFNK
FLKYQQQQQGGSQSQKGKQQEEENEGSNILSGFAPEF	NLGQSQVRQL	VSIDAKDFHL
FLQQTGEKVKGAAQGATEAVKQTLGLGEHDQDNRRNY	NLGQSQVRQLK	VTRGQGKVRVV
FLVPPQESQR	NLHPGDAQRIPAGTTY	VVAEQAGEQGFE
FNTNEDIAEKLQSPDDERK	NLRQSQVSELKYEGNWGPLVNPESQQGSP	VVLPVQNDGSTGLHW
FNTNEDTAEKLRSPDDERK	NLRQSQVSELKYEGNWGPLVNPESQQGSPV	RVQSRNELDKGIGTIISSPYR
FNTNEDTAEKLRSPDDERKQIVTVEGGL	NLRSRDPIYSNKLGKF	VVTTGRKIIGATNPAQSEPGTIRGDF
FNYPSEMVNGVFDRK	NNENLRLIT	WILGDVFIR
FPEQNYIVKEGQLASQYR	NNNPFKFLVPPQESQKR	WRKKGVITQ
FPGSAQAVEK	NNNPFSFLVPPKESQRR	YKNGIYSPHW
FPRPQPRQEEEHEQR	NNPFKFLVPPQESQKR	YLAGNPDIEHPET

FSKHFLAQSFNTNEDTAEK	NNQLDQNPRVFY	YLAGNPDIEHPETM
FSLKAGVKPH	NNQLDQTPRVF	YLVNPHDHQNL
FSREEGQQQGEQRLQESV	NNQLDQTPRVFY	YLVNPHDHQNLKI
FSRNILEASY	NPFKFLVPPQESQKR	YRNGIYSPH
FSVDKQIAKNLQGENEGEDKGAIVTVKGGL	NPFKFLVPPQESQKRA	AIHATQGSTQQLGPLVTVPQFL
FTPLTVTPQGEYNVR	NPFLFGSNRFETL	ALEPDHRVESEGGL
FTPLTVTPQGEYNVRV	NQLDQNPRV	ERKQIVTVEGGL
FTPLTVTPQGEYNVRVS	NRIESEGGF	FKNQYGHVR
FVTAVVGFGKKRGPN	NSKPNTLLLPHH	FKNQYGHVRV
FVTAVVGFGKKRGPNSSEK	NSKPNTLLLPHHAD	FNLKSQQAR
FYLAGNQEQEFLK	NSKPNTLLLPNHAD	FVDAQPQQKEEGSKGR
GFSKHFLAQSFNTNE	NSLTLPALRQF	FVDAQPQQKEEGSKGRKG
GFSKHFLAQSFNTNEDIAEK	NSLTLPALRQFGLSAQ	FVDAQPQQKEEGSKGRKGP
GGIGEKREQKPLG	NSLTLPALRQFGLSAQY	GALLLPHF
GGIGEKREQKPLGSIIGESF	NVISQIPSQVQELAFPGSAQAVEKLLKNQRESY	HLAIPVNKPGRFESF
GGLSVIKPPTD	NVTVPKVEKKPE	KGAIVTVKGGL
GGLSVISPKW	NWGPLVNPESQQGSPR	KLLKNQRESY
GKQQEEENEGSNILSGFAPEF	PALRQFGLSAQY	KTISSEDKPFNLR
GKREQDQDQDEDEDEDEDQPRKSREWRS	PALRQFQLSAQ	KTNDRPSIGNLAGA
GQHQQEEEEEGGSVLSGFSKHF	PALSWLKL	KTNDRPSIGNLAGAN
GQHQQEEEEEGGSVLSGFSKHFL	PALSWLRLSAE	KTNDRPSIGNLAGANSL
GQHQQEEEEEGGSVLSGFSKHFLAQSF	PDNRIESEGGF	KYEGNWGPLVNPESQQGSPR
GQHQQEEEEEGGSVLSGFSKHFLAQSFNTNE	PFKFLVPPQESQKR	LAFPGSAQAVEK
GQHQQEEEEEGGSVLSGFSKHFLAQSFNTNED	PFSFLVPPQESQR	LAFPGSAQDVERL
GQHQQEEEEEGGSVLSGFSKHFLAQSFNTNEDIAEK	PGSAQAVEKLLKNQRESY	LAIPVNKPGRFESF
GQHQQQEEEGGSVLSGFSKHFLAQS	PIYSNKLGK	LDTSNFNNQLDQTPR
GQHQQQEEEGGSVLSGFSKHFLAQSF	PIYSNKLGKF	LKYQGNSGPLVNP
GQHQQQEEEGGSVLSGFSKHFLAQSFNTNE	PIYSNKLGKL	LLKNQRESY
GQHQQQEEEGGSVLSGFSKHFLAQSFNTNEDTAEK	PIYSNNFGKF	NALEPDHRVE
GQIPRPRP	PLDLTSFVLH	NKRSQQLQNLRD
GRKQGQHQQEEEEEGGSVLSGFSKHFLAQSFNTNE	PLVNPESQQGSPR	NQRSPQLQNLRD
GSAQAVEKL	PLVVQFQKFRSS	RAIPSEVL
GSAQAVEKLLKNQR	PLVVQFQKLDKESL	RKQIVTVEGGL
GSIFAISR	PMIGTLAGANSLL	VEKLLKNQRESY
GVEENICTLK	PPGVPYWTYNTGDEPVVA	VIQHTFNLKSQQAR
GVFTFEDETTSPVAPATLYK	PPNPHIGINVNSIR	VIQHTFNLKSQQARQ
GVFTFEDETTSPVAPATLYKA	PQHHTNPTKPINL	VPIPQHHTNPTKPINL
GVIGSMFKAVQDTYENAK	PQLENLRD	VRQLKYQGNSGPLVNP
GVLNVTVPKVEKKPENKNVRQIDVV	PQLQNLRD	VTPTKPINL
HAIATGDLVSLSEQEL	PSEVLAHSY	YEGNWGPLVNPESQQGSPR
HFLAQSFNTNE	PSIGNLAGANSLL	AIGINAENNQRNF
HFLAQSFNTNED	PVHAASVTY	AIPVNKPGRF
HFLAQSFNTNEDIAEK	QIKNNNPFKFLVPPQESQKR	ALRVPAGTTY
HFLAQSFNTNEDTAEK	QIVRNLQGENEEEDSGA	ALRVPSGTTY
HTEEGIKSLDQFLSGK	RAELSEDDV	AQRIPAGTTY
HTFNLKSQQAR	RAELSEDDVF	AVEKLLKNQRESY

IAEKLQSPDDER	RAELSEQDI	FAFGINAENNQRNF
IAEKLQSPDDERKQ	RAIPSEVLAH	FAIGINAENNQRNF
IAEKLQSPDDERKQIV	RAIPSEVLAHSY	FEITPEKNPQLRD
IAEKLQSPDDERKQIVTVEGGL	RAIPSEVLAHSYNLRQ	FLAPIDTKPQTHAGY
IAGASDKPVDESDAAAIQA	RAIPSEVLAHSYNLRQSQVSE	FPFELPRE
IAGASDKPVDESDAAAIQAAE	RAIPSEVLAHSYNLRQSQVSELKYEGNWG[illegible]	[illegible]QQGSPR
IAGASDKPVDESDAAAIQAAEM	RDPIYSNKLGKF	IIYALNGRAL
IEAAHAIATGDLVSL	RGIPNSISI	IQKLNALKPDNRIESEGGF
IEKNRDKPAQQEQGKDEQEQGKDEL	RLQSGDALRVPSGTTY	IRHFNEGDVL
IENLIKSQSESYFVDAQPQQKEEGN	RMITLAIPVNKPGRFESF	ISKEQIRAL
IERQVQELAFPGSAQDVERL	RPSIGNLAGANSLLNALPEEVIQHTF	ISKEQIRALSK
IERQVQELAFPGSAQDVERLLK	RQFQLSAQY	ISKEQIRALSKR
IGINAENNQR	RQIKNNNPF	ISKKQIRELSK
IGINAENNQRNFL	RQLKYQGNSGPLVNP	ITLAIPVNKPGRF
IGINAENNQRNFLAG	RQNIGQNSSPDIYNPQAG	LAIPVNKPGRF
IGINAENNQRNFLAGSQD	RSPQLENLRD	LAPIDTKPQTHAGY
IGINAENNQRNFLAGSQDNVISQIPSQVQE	RTPLMQVPVL	LARPVLGGSSTFPYPR
IGKAKSDALFKAVE	SAEFGSLRKNAM	LHIPVSVSTPGKF
IKEKLPGGHSDK	SAFSWNVLQAALQTPKGKL	LRVPAGTTY
IKEKLPGHHTH	SAQAVEKLLK	LRVPSGTTY
IKNQRESYFADAQPLQKEEGKKG	SAQAVEKLLKNQRESY	LVPPQESQKRA
ILQGDAIVTSSGK	SAQDVERL	LVQKGLPRNTQGVAGLGHAPISLPNQL
ILQISGERVK	SFFFPFELPREER	MQIVRNLQGENE
ILQISGERVKE	SFLVPPKESQRR	MQIVRNLQGENEE
INAENNQRNFLAGSKDNVISQIPSQVQE	SFLVPPQESQR	NLRQSQVSEL
INAENNQRNFLAGSQDNVISQIPSQVQE	SGAIVTVKGGL	NNQLDQNPRV
INANGIIETTFLPSK	SGDALRVPSGTTY	PGITIGDTIPDL
INAYPSVDLVMDKPNGPVWRIS	SGFAPEFLKEAFGVN	RFYLAGNQEQEF
IPVNKPGRFESFF	SGGIKLPTDIISKISPLPVL	RIESEGGF
IQKLNALK	SHQKIRHFNEGDVL	RNGLHLPSYSPYPRM
IQKLNALKPDNRIES	SILSGFAPEFLEHA	RSRDPIYSNKLGKF
ISQIPSQVQELAFPGSAQAVEK	SKEQIRELSKRAKS	SKRPTISNGY
ITAGKDTPQGSIE	SKHFLAQSF	SLLNALPEEVIQHTFNLK
ITAGKDTPQGSIEALQAGE	SKHFLAQSFNTNED	SLVNNDDRDSY
ITEAPNAPVTAQR	SKKLETYNIFEHD	SRDPIYSNKL
IVEISKEQIRALS	SKPNTILLPHHADADF	SRDPIYSNKLGKF
IVGSTSGGTMISTSTPHMVL	SKPNTLLLPNHAD	TLNRNGLHLPSYSPYPRM
IVPPSKTTAGILLPEK	SKRPTISNGYGRLTEVGPDDDEKSWLQRL[illegible]	TLVNNDDRDSYNLHPGDAQRIPAGTTY
IVRNLQGENEEE	SLLNALPEEVIQHTF	VDLNGNHL
IVRNLQGENEEEDSGAIVTVKGGL	SLLNALPEEVIQHTFNLKSQQAR	VMDKPNGPVWRISGEDL
IVRNLQGENEEEDSGAIVTVKGGLR	SPQLENLR	VVNPDNNENLRL
		YVVNPDNDENLRM
IVTSSGKL	SPQLENLRD	AAHAIATGDL
KAADQIAGQTF	SPQLQNLRD	AGANSLLNAL
KAADQIAGQTFNDVGRF	SQDNVISQIPSQVQELAFPGSAQAVEKLLKNQRESY	AQQLPKQAQVK
KAADQIAGQTFNDVGRFDEEE	SQKQQLQDSHQKIR	DIAEKLQSPDDERKQIVTVEGG
KAAGSTTAQYVGEK	SQKQQLQDSHQKIRHF	FAIGINAENNQRN

KAGMEKTKATVQEKAER	SQQARQVKNNNPFSF	FNKRSQQLQNLRD
KAHIFRADLPGVK	SQQLQNLRD	IIIAQGKGAL
KAHIFRADLPGVKKEDLK	SRDPIYSNKLGK	KLQSPDDERKQIVTVEGGL
KAHIFRADLPGVKKEDLKVQVEENK	SRKTISSEDKPFNLR	LKYEGNWGPLVNPESQQGSPR
KAIENVPDLKSYLGAR	SSPRGKVEAEKARPM	NQRSPQLQNL
KAKAAGSTTAQYVGEK	SSSRKTISSEDKPF	QIKNNNPFK
KALQKIME	SSSRKTISSEDKPFNLR	QIVRNLQGEN
KATAVGWAAAHFSAEK	SSSRKTISSEDKPFNLRSRD	RVTAPAMRKPQQEEDDDDE
KDATLEKGQQGYAVTK	SVISPKWQEQQ	SRYPTSKGAIIFGDAPNN
KDYGSYAQGRINE	SVIVEISKEQIRALSKRAK	SVIKPPTDEQQQRPQE
KEAKMNQAEL	SVIVEISKKQIRELSKHAK	SVIKPPTDEQQQRPQEE
KEAKMSQAELDKQAARQHNTAA	SVLSGFSKHF	VRLVLPGEL
KEAKMSQAELDKQAARQHNTAAK	SYFVDAQPKKKEEGN	
KEDLKVQVEENK	SYFVDAQPQQKEEGSKGRKG	
KELAFNYPSEMVNGVFDRK	SYNLQSGDALRVPAG	
KELQEFKPAGDAPSFTSADL	SYRLQSGDALRVPSG	
KELQEFKPAGDAPSFTSADLGVK	TAEKLRSPDDERKQIVTVEGGL	
KETEVEEEDPEL	TEMKLRPPFP	
KEVGQKTKEVGQDIQSK	TGLGELGEDVL	
KGKQQEEENEGSNILSGFAPEF	TISSEDEPFNLR	
KHFLAQSFNTNE	TISSEDKPFNLR	
KHFLAQSFNTNEDIAEK	TLEFLEHAFSVDK	
KIDKHMYHDMY	TLTAFLGRSVSL	
KKGILDKIKEKLPGGHSDK	TLVNNDDRDSY	
KKQSGPASTEIKSADEATAFIGENK	TLVNNDDRDSYNLHPGDAQRIPAGTTYY	
KLGGQHRDAEL	TPKPSSLGRAL	
KLLKNQRESYFVDAQPKKKEEGN	TPLMQVPVL	
KLNALKPDNRIES	TPMIGTLAGANSLLNALPEE	
KLNPYAKTAKR	TPVVAVSIIDTNSLENQLDQMPRRF	
KLPADKAATLQ	TRRLVVSKNKPLVVQF	
KNQRESYFVDAQP	TRRLVVSKNKPLVVQFQKLDKES	
KNQRESYFVDAQPKK	TSNFNNQLDQNPRVFY	
KNQRESYFVDAQPKKKEEGN	TSNFNNQLDQTPRV	
KPENKNVRQIDVV	TSNFNNQLDQTPRVF	
KQGQHQQEEEEEGGSVLSGFSKHFLAQSFNTNE	TTYYVVNPDNNENLRL	
KQGQHQQEEEEEGGSVLSGFSKHFLAQSFNTNEDIAEK	TVEGTKAAAHVVEGAAGY	
KQKSQELKDKLGGQHRDAEL	TVTVPKEEDKKPQVKSIQISA	
KQKSQEVKEKLGGQHRDAEL	VATVSLPR	
KQQEEENEGSNILSGFAPEF	VEISKKQIR	
KQRESYFVDAQPQQKEEGSKGRKG	VEKLLKNQRE	
KQTDEYGNPVHAASVT	VGEKAVQAKDVAVESGKSAAGY	
KQTDEYGNPVHAASVTYVATR	VGLNKGHVVTKK	
KQVKELAFPAGSAQDIENL	VIQHTFNLKSQQA	
KRAVGYLSGK	VLAIHATQGSTQQLGPLVTVPQFL	
KREQDQDQDEDEDEDEDQPRKSREW	VLQRFNKR	
KRQQEEENEGGSILSGFAPEF	VQELAFPGSAQAVEKLLKNQRESY	

KSDALFKAVE	VRLVLPGELAK	
KSHGGRKQGQHQQQEEEGGSVLSGF	VSIIDTNSLENQLDQMPR	
KSQSDNFEYVSFK	VSIIDTNSLENQLDQMPRRF	
KSSWWPFGGESK	VSIIDTNSLENQLDQMPRRFY	
KTNDRPSIGNLAGANSLLNAL	VSIIDTNSLENQLDQMPRRFYL	
KTNDRPSIGNLAGANSLLNALPEE	VVAARSQSDNFE	
KTNDTPMIGTLAGANSL	VVAARSQSDNFEY	
KVAADLRDKA	VVAARSQSDNFEYVSF	
KVEAEKARPMEETK	VVDLKTSLPEWVRIGF	
KVEAEMDLEAK	VVFKTHHNAVSSYIKDVF	
KYEGNWGPLVNPE	VVLFNIKGRAVLGL	
LAAGANGGSAGKL	VVLPVQNDGSTGLHWANLQKR	
LAEAAEYVGQK	VVNPDNDENLRM	
LAFGINAENNQRNFLAGEK	VVNPDNDENLRMIT	
LAFPGSAKDIEN	VVNPDNNENLRLIT	
LAFPGSAKDIENLIKS	WQVKSGTLFDNVL	
LAFPGSAKDIENLIKSQ	YAKGYLTDK	
LAFPGSAQAVEKLL	YLAGNQEQEFLK	
LAFPGSAQAVEKLLKNQR	YLRGSQISSNNFGKF	
LAFPGSAQAVEKLLKNQRES	YNNEDTPVVA	
LAFPGSAQAVEKLLKNQRESYFVDAQPKK	YNPKAGRISTL	
LAFPGSAQDVERLL	YRILEFNS	
LAGEKDNVVRQ	YRILEFNSKPN	
LAGNQEQEFL	YRILEFNSKPNTLLLPHHAD	
LAGNQEQEFLK	YRILEFNSKPNTLLLPNH	
LAGSKDNVISQIPSQVQE	YRIVQFQSKPN	
LAQSFNTNEDIAEKLQSPDDERKQIVTVEGGL	YRIVQFQSKPNTILLPHHAD	
LAQSFNTNEDTAEK	YRIVQFQSKPNTILLPHHADAD	
LARPVLGGSSTFPYPRR	YRIVQFQSKPNTILLPHHADADF	
LARPVLGGSSTFPYPRRG	YSNKLGKF	
LATGWSAAH	YVSFKTNDRP	
LDQDKVKLAD	YVSFKTNDRPSIG	
LDTSNFNNQLDQNPRVF	YVSFKTNDRPSIGNL	
LDTSNFNNQLDQNPRVFYL	YVSFKTNDRPSIGNLA	
LDTSNFNNQLDQTPRVFYL	YVSFKTNDRPSIGNLAGANSLLNALPEE	
LDTSNFNNQLDQTPRVFYLAGNPDIEYPETMQQQQQQKSHGGR	YYVFKTHHNAVSSY	
LEAKGREKAETKEGGGEEKGMR	YVVNPDNDENLRMIT<br>YVVNPDNNENLRLIT	
LEEIFKYRAEA	AAAATLNQNRQVTDA	
LEEKQKKKMEKELINL	AAEMERESL	
LEHAFSVDK	AAHAIATGDLVSL	
LEHAFVVDRQ	AIHATQGSTQQLGPLVTVPQF	
LGEDVLAIH	AKAAGSTTAQYVGEK	
LGEDVLAIHA	ALKPDNRIE	
LGQDVLAIHA	ALPEEVIQHTF	
LHENIARPS	ALPEEVIQHTFNLK	
LIREVAGFAPYEKRITEL	ALPEEVIQHTFNLKSQQAR	

LKAIENVPDLKSYLGAR	AQSFNTNEDIAEK	
LKDISKYRAEA	ASHRQSYEAGQTKGRTEEK	
LKEAFGVNMQIVRNLQGE	ASNLPHASSNIDPLDLTSF	
LKNQRESYFVDAQPKK	DERKQIVTVEGGL	
LKNQRESYFVDAQPKKKEEGN	DIAEKLQSPDDERK	
LKVGKDKRALK	DKIKEKLPGGHSDK	
LKVQVEENK	DQTPRVFYL	
LKYEGNWGPLVNPESQQGSPRV	DQTPRVFYLA	
LKYEGNWGPLVNPESQQGSPRVK	DRPSIGNLAGANSLL	
LLKNQRESYFVDAQPKK	DTAEKLRSPDDERK	
LLKNQRESYFVDAQPKKKEE	EQVRALAPTKK	
LLKNQRESYFVDAQPKKKEEGN	ESFFFPFELPREER	
LNALPEEVIQH	ESFFFPFELPREERG	
LNALPEEVIQHT	ESLFFPFELPSEERGR	
LNALPEEVIQHTF	ESYFVDAQPKKKEEGN	
LNALPEEVIQHTFNLKSQQAR	FADAQPLQKEEGKKG	
LNALPEEVIQHTFNLKSQQARQ	FKAVQDTYENAK	
LNNLNALEPDHRVES	FLEHAFSVDK	
LPEGPAVKIGENKD	FLVPPQESQKR	
LPSKKEYLRL	FNSKPNTLLLPHHAD	
LPSKKEYLRLT	FNTNEDIAEK	
LQEFKPAGDAPSFTSADLGVKK	FSKHFLAQSFNTNE	
LQEKVHDPAGKGGPVFGAGK	FTPLTVTPQGEYNVRVSS	
LQEKVHDPAGKGGPVFGAGKDEDKQDLGVTGTG	GARQLEENL	
LQGFSKNILEASYDTK	GNWGPLVNPESQQGSPR	
LQGFSRNIL	GPLVNPESQQGSPR	
LQGFSRNILEASYDTK	HAIATGDLVSL	
LQLNKVDENGTPKPSSLGRAL	HHTNPTKPINL	
LQSPDDERKQIVTVEGGLS	IAEKLQSPDDERK	
LQSPDDERKQIVTVEGGLSVISPKWQEQQ	IQHTFNLKSQQAR	
LQTPKGKLEKLFDQQNEGSIFAISR	IVEISKEQIR	
LRSPDDERKQIV	IVEISKEQIRALSK	
LVMDKPNGPVWRIS	IVKEGQLASQYR	
LVVLPVQNDGSTGLH	KAHFTAPSSVR	
MDWIGAYR	KEAGAKMELHEAKAR	
MESQQANREEL	KFLVPPQESQKR	
MESQQANREELDEK	KIKEKLPGGHSD	
MFKAVQDTYENAK	KIKEKLPGGHSDK	
MIYDVNSPLFR	KPLVVQFQKLDKES	
MIYDVNSPLFRS	KQIVTVEGGL	
MIYDVNSPLFRSF	KQTDEYGNPVHAA	
MKVKGNVFKNKR	KQTDEYGNPVHAASVTY	
MLGGLLHRG	LAFPGSAK	
MPGLLWGASK	LAFPGSAKDIENLIK	
MQGGKKAGESIKE	LAFPGSAQAVEKL	
MQGGKKAGESIKETAANIGASA	LAFPGSAQAVEKLLK	

MQIVRNLQGENEEEDSGAIVTVKGGL	LAFPGSAQAVEKLLKN	
MTARDPVQKELATQK	LAFPGSAQAVEKLLKNQ	
MVNGVFDRK	LAFPGSAQAVEKLLKNQRE	
NALKPDNRIE	LAFPGSAQAVEKLLKNQRESY	
NALPEEVIQHTFNLKSQQA	LAFPGSAQDVERLLK	
NEEEDSGAIVTVK	LAQSFNTNEDIAEK	
NEEEDSGAIVTVKGGL	LDTSNFNNQLDQTPRVF	
NEEEDSGAIVTVKGGLR	LDTSNFNNQLDQTPRVFY	
NEGEDKGAIVTVKGGL	LEHAFSVDKQIAKNLQGENEGEDKGAIVTVKGGL	
NLKTENETQDNDQKGYGGAK	LEHAFVVDR	
NNNPFKFLVPPQ	LKEAFGVNM	
NNPFSFLVPPQESQRR	LKEAFGVNMQ	
NQHSVFPLN	LLKNQRESYF	
NQRESYFVDAQPKK	LNALPEEVIQHTFNLK	
NQRNFLAGSK	LQSPDDERKQIV	
NQRNFLAGSQDNVISQIPSQVQE	LQSPDDERKQIVTVEGGL	
NQRSPQLQNLR	LRSPDDERKQIVTVEGGL	
NQSEQLEGKEKKQMER	LVLPVQNDASTGLH	
NQSEQLEGKEKKQMEREL	LVPPQESQKR	
NSNKINAYPSVDLVMDKPNGPVWRISG	LVPPQESQR	
NTNEDIAEKLQSPDDERK	LVPPQESQRRA	
NTNEDIAEKLQSPDDERKQIVTVEGGL	LVVGEVEVSK	
NTNEDTAEKLRSPD	MDWIGAYRG	
NTNEDTAEKLRSPDDERK	MLGGLLHR	
NTRAGGNPNDYGYGTGGTYN	MQIVRNLQGENEEEDSGAIVT<br>MQIVRNLQGENEEEDSGAIVTVKGG<br>LR	
NTTTEKEIHVEK	NALEPDHRVESEGGL	
NVISQIPSQ	NALPEEVIQHTFNLK	
NVTVPKVEKKPENKNVRQ	NALPEEVIQHTFNLKSQQAR	
NVVKGVAEYAGQK	NVTVPKVEKKPENKNVR	
PDNRIESEGGFIE	PALSWLRL	
PEEVIQHTFNLKS	PEEVIQHTF	
PEEVIQHTFNLKSQ	PEEVIQHTFNLK	
PEEVIQHTFNLKSQQ	PEEVIQHTFNLKSQQAR	
PEEVIQHTFNLKSQQARQ	RADLPGVKKEDLK	
PEEVIQHTFNLKSQQARQVKNNNPFSFLVPPQESQR	RAIPSEVLAHSYNLRQSQ	
PEEVIQQTF	RGHKIKGTVVL	
PEEVIQQTFNLR	RPSIGNLAGANSL	
PLDLTSFVLHEA	RPSIGNLAGANSLL	
PLDLTSFVLHEAI	RPSIGNLAGANSLLNALPEE	
PQHHTNPTKPINLLVLP	RQFQLSAQ	
PVQNDASTGLHWANL	SGAIVTVKGGLR	
QESVIVEISK	SGFSKHFLAQSFNTNE	
QEVRVQDKEYGTGRGTGEK	SGIIHKIEETL	
QIAGQTFNDVGRF	SKHFLAQSFNTNE	
QIAKNLQGENEGEDK	SKHFLAQSFNTNEDIAEK	
QIAKNLQGENEGEDKGAIVTVK		

QIKNNNPFKFLVPPQESQK<br>QIPSQVQELAFPGSAQAVEK<br>QIVRNLQGE<br>QIVRNLQGENEEE<br>QIVRNLQGENEEED<br>QIVRNLQGENEEEDSG<br>QIVRNLQGENEEEDSGAIV<br>QIVRNLQGENEEEDSGAIVT<br>QIVRNLQGENEEEDSGAIVTVK<br>QIVRNLQGENEEEDSGAIVTVKGGLR<br>QQQEEQPLEVRK<br>QSFNTNEDIAEK<br>QVKNNNPFSFLVPPQESQRR<br>QVKNNNPFSFLVPPQESQRRA<br>RAELSEQDIFVIPA<br>RDKATAVGWAAAHFSAEK<br>REEIGEENKQSSLEE<br>RENTTTEKEIHVEK<br>REQDQDQDEDEDEDEDQPRKSREWRSK<br>RERQQHGEKEEDEGEQPRPFP<br>RFVTAVVGFGKKRGPN<br>RFVTAVVGFGKKRGPNSSEK<br>RFYLAGNQEQE<br>RFYLAGNQEQEFL<br>RGPGGTATAHNTRAGGNPNDYGYGTGGTYN<br>RGVHVDKFEEKK<br>RPSIGNLAGANSLLNAL<br>RQFPFPRPPH<br>RQFPFPRPPHQKEER<br>RQIKNNNPFK<br>RQIKNNNPFKFLVPPQESQKR<br>RQIKNNNPFKFLVPPQESQKRA<br>RQTDEYGNPVHAT<br>RQTDEYGNPVHATSVTY<br>RQTDEYGNPVHATSVTYVA<br>RQTDEYGNPVHATSVTYVATK<br>RQVKNNNPFSFLVPPKESQRR<br>RQVKNNNPFSFLVPPQESQRR<br>RQVQELAFPGSAQDVERL<br>RQVQELAFPGSAQDVERLL<br>RQVQELAFPGSAQDVERLLK<br>RRGQLLVVPQNFVVAEQGGEQGLE<br>RSQQLQNLR<br>RSSNSFQTL<br>RVLGKTQPGGASSVMQSA<br>RVLMSAPLSAEL	SKHFLAQSFNTNEDTAEK<br>SPDDERKQIVTVEGGL<br>SQLLDKAKNFVSEK<br>SQVRQLKYQGNSGPLVNP<br>STIVGSSGGTMISTSTPHMVL<br>TAVGWAAAHFSAEK<br>TFNLKSQQAR<br>TKDYASDATDAAKK<br>TLEFLEHA<br>TNDRPSIGNLAGANSLL<br>TPMIGTLAGANSLL<br>VIQHTFNLK<br>VSELKYEGNWGPLVNPESQQGSPR<br>VVGEVEVSK<br>VVLPVQNDGSTGLHWANLQK<br>YVVNPDNNENLRL<br>AIHATQGSTQQLGPLVTVPQFL<br>ALEPDHRVESEGGL<br>ERKQIVTVEGGL<br>FKNQYGHVR<br>FKNQYGHVRV<br>FNLKSQQAR<br>FVDAQPQQKEEGSKGR<br>FVDAQPQQKEEGSKGRKG<br>FVDAQPQQKEEGSKGRKGP<br>GALLLPHF<br>ITLAIPVNKPGRFESF<br>KGAIVTVKGGL<br>KLLKNQRESY<br>KTISSEDKPFNLR<br>KTNDRPSIGNLAGA<br>KTNDRPSIGNLAGAN<br>KTNDRPSIGNLAGANSL<br>KYEGNWGPLVNPESQQGSPR<br>LAFPGSAQAVEK<br>LAFPGSAQDVERL<br>LAIPVNKPGRFESF<br>LDTSNFNNQLDQTPR<br>LKYQGNSGPLVNP<br>LLKNQRESY<br>NALEPDHRVE<br>NKRSQQLQNLRD<br>NQRSPQLQNLRD<br>RAIPSEVL<br>RKQIVTVEGGL<br>VEKLLKNQRESY<br>VIQHTFNLKSQQAR	

RVTAPAMRKPQ	VIQHTFNLKSQQARQ	
SAADAAKRAVGYLSGK	VPIPQHHTNPTKPINL	
SAAGYAAKVAADLRD	VRQLKYQGNSGPLVNP	
SAAGYAAKVAADLRDKA	VTPTKPINL	
SAQAVEKLLKNQR	YEGNWGPLVNPESQQGSPR	
SDKEDSVFKGINS	AIGINAENNQRNF	
SDKEDSVFKGINSYR	AIPVNKPGRF	
SEQDIFVIPA	ALRVPAGTTY	
SERSAWEQISNYSDQA	ALRVPSGTTY	
SFDYEPFYLR	AQRIPAGTTY	
SFDYEPFYLRG	AVEKLLKNQRESY	
SFDYEPFYLRGS	FAFGINAENNQRNF	
SFDYEPFYLRGSQ	FAIGINAENNQRNF	
SFNTNEDIAEK	FEITPEKNPQLRD	
SGFAPEFLEHA	FLAPIDTKPQTHAGY	
SGIIHKIEE	FPFELPRE	
SGIIHKIEET	IIPLPVIKE	
SIIDKIKEKFQ	IIYALNGRAL	
SKDYAGDTAQKTKDYAGDTAQK	IQKLNALKPDNRIESEGGF	
SKKLETYNIFEHDPDFKNPIGWSTAVTK	IRHFNEGDVL	
SKNILEASYDTK	ISKEQIRAL	
SKRPTISNGYGRL	ISKEQIRALSK	
SLFFPFELPSEERGR	ISKEQIRALSKR	
SNSYNLGQSQVRQLK	ISKKQIRELSK	
SNSYNLGQSQVRQLKYQGNSGPLVNP	ITLAIPVNKPGRF	
SQDNVISQIPSQVQELAFPGSAQAVEK	LAIPVNKPGRF	
SQEQLKKPQGEQDPIKYGDVFKVSD	LAPIDTKPQTHAGY	
SQKGKQQEEENEGSNILSGFAPEF	LARPVLGGSSTFPYPR	
SQLLDKAKN	LHIPVSVSTPGKF	
SQQARQVKNNNPFSFLVPPQESQRR	LRVPAGTTY	
SQVSELKYEGNWGPLVNPESQQGSPR	LRVPSGTTY	
SRYPTSKGAIIFGDAPNNMRQFQNQD	LVPPQESQKRA	
SRYPTSKGALIFGDAPNNMQQFHNQDIFHDLA	LVQKGLPRNTQGVAGLGHAPISLPNQL	
SSDFLTYGLK	MQIVRNLQGENE	
STIVGSSGGTMISTSTPHMVLQQSL	MQIVRNLQGENEE	
STIVGSTSGGTMISTSTPH	NLRQSQVSEL	
STIVGSTSGGTMISTSTPHMVL	NNQLDQNPRV	
STIVGSTSGGTMISTSTPHMVLQ	PGITIGDTIPDL	
STLDTLTAFLGRS	RFYLAGNQEQEF	
SVAAAARLNENVK	RIESEGGF	
SVDHITTPFIVE	RNGLHLPSYSPYPRM	
SVDHITTPFIVEA	RSRDPIYSNKLGKF	
SVDHITTPLAEK	SKRPTISNGY	
SVDTAKGLAAAAAGENAKEYTARK	SLLNALPEEVIQHTFNLK	
SVGGYNDDTNKQHDTTGVYPEK	SLVNNDDRDSY	
SVGHMPSTKEEGHDFQEAK	SRDPIYSNKL	

SVIKPPTDEQQQRPQ	SRDPIYSNKLGKF	
SVIKPPTDEQQQRPQEEEEEEEEDEK	TLNRNGLHLPSYSPYPRM	
SVISPKWQEQ	TLVNNDDRDSYNLHPGDAQRIPAGTTY	
SVISPKWQEQEDEDEDED	VDLNGNHL	
SVISPKWQEQQDEDEDEDEDDEDEQIPSHPPRRPSH	VMDKPNGPVWRISGEDL	
SVISPKWQEQQDEDEDEDEDDEDEQIPSHPPRRPSHG	VVNPDNNENLRL	
SYFVDAQPKK	YVVNPDNDENLRM	
TADNKTGSKVGEYADYGSQK		
TAIGETVSSAGEK		
TAVGWAAAH		
TAVGWAAAHF		
TDASATVPIPDVMK		
TEAGEDTKEEGVHDEL		
TEETKEKVSETAEATANKAAEMK		
TEVGPDDDEKSWLQRL		
TEVGPDDEKSWLQRL		
TFRGIPNSISI		
TGSKVGEYADYGSQK		
TGSKVGEYADYGSQKA		
TIQQRSNSLVPYELHEVADAK		
TKAATNVVKGVAEYAGQK		
TKDYASDATDAAKKT		
TKDYASDATDAAKKTKDYAAQK		
TKDYASEASDVAQNTKDYAAQK		
TKDYATDAAQKTKDYATQK		
TKEGKDATVNKMGEYKDYTAEK		
TKEVGQKTKEVGQDIQ		
TKEVGQKTKEVGQDIQSK		
TKEVGQKTKEVGQDIQSKAQDTR		
TLKAADQIAGQTFNDVGRFDEEE		
TMDYATDAAQK		
TNDRPSIGNLAGANSL		
TNDRPSIGNLAGANSLLNAL		
TNDRPSIGNLAGANSLLNALPEE		
TNDTPMIGTL		
TNRGPGGTATAHNTRAGGNPNDYGYGTGGTYN		
TPMIGTLAGA		
TPMIGTLAGANSL		
TSVGHMPSTKEEGHDFQEAK		
TSVGHMPSTKEEGHDFQESK		
TVEGTKAAAHVVEGAAGYAGHK		
TVEGTKAAAHVVEGAAGYAGHKAA		
TVEGTKAAAHVVEGAAGYAGHKAAEL		
TVEGTKAAAHVVEGAAGYAGHKAAELASM		
TVSFSWNKFVPKQPNMILQGDAIVTSSGK		
TVTVPKEEDKKPQVK		

TYLPSETPAPLVKYRE<br>TYLPSETPAPLVKYREEE<br>VAADLRDKATAVGWAAAHFSAEK<br>VAAKELEQL<br>VAIVGVFPK<br>VAKELAFNYPSEMVNGVFDRK<br>VARNEGVSVTETK<br>VAVESGKSAAGYAAK<br>VAVESGKSAAGYAAKVAADLRD<br>VDHITTPFIVE<br>VEAKGREKDVTKEGGVEEQGRGR<br>VEESRPGAIAE<br>VEESRPGAIAETL<br>VEESRPGAIAETLK<br>VEGGLSVISPK<br>VEKLLKNQR<br>VESSQGAFQYFGLK<br>VGHMPSTKEEGHDFQEAK<br>VGHMPSTKEEGHDFQESK<br>VGLNKGHVVTK<br>VGWAAAHFSAEK<br>VHGAPIYDSA<br>VIQHTFNLKSQQ<br>VIQHTFNLKSQQARQIKNNNPFKFLVPPQESQKR<br>VIQQTFNLR<br>VKGAAQGATEAVKQTLGLGEHDQDNRRNY<br>VKKPLDKATEGGREVL<br>VKLADVLTGATA<br>VKNNNPFSFLVPPQESQRR<br>VKRTTVTATTATA<br>VLAHSYNLRQSQ<br>VLSNSYNLGQSQVR<br>VMDKPNGPVWRIS<br>VMDKPNGPVWRISG<br>VNSPGTGVVDKL<br>VPIPQHHTNPTKPINLLVLPVQND<br>VPIPQHHTNPTKPINLLVLPVQNDASTGLH<br>VRATGSNVITPG<br>VRATGSNVITPGGLA<br>VRATGSNVITPGGLAA<br>VRATGSNVITPGGLAAM<br>VRATGSNVITPGGLAAMA<br>VRLVLPGELA<br>VRLVLPGELAKHAVSEGTKAVTKFTSS<br>VSELKYEGNWGPLVNPE<br>VSLKLQKR		

VSLKLQKRLAA<br>VSLKLQKRLAASV<br>VTPTKPINLVVLPVQNDGSTGLHWANL<br>VVGFGKKRGPN<br>VVKGVAEYAGQK<br>VVLPVQNDGSTGLHWAN<br>VVLPVQNDGSTGLHWANL<br>YEGNWGPLVNPESQQGSPRV<br>YFVDAQPKK<br>YFVDAQPKKKEEGN<br>YIRETQPASENLAAAAKHHLAEAAEYVGQK<br>YQAFTQVF<br>YQTKVTDPTGAGGAEIDITPVEKSFSR<br>YTAKKKEEAQREL<br>YTIGSVDHITTPFIVE<br>AAAATLNQNRQVTDA<br>AAEMERESL<br>AAHAIATGDLVSL<br>AIHATQGSTQQLGPLVTVPQF<br>AKAAGSTTAQYVGEK<br>ALKPDNRIE<br>ALPEEVIQHTF<br>ALPEEVIQHTFNLK<br>ALPEEVIQHTFNLKSQQAR<br>AQSFNTNEDIAEK<br>ASHRQSYEAGQTKGRTEEK<br>ASNLPHASSNIDPLDLTSF<br>DERKQIVTVEGGL<br>DIAEKLQSPDDERK<br>DKIKEKLPGGHSDK<br>DQTPRVFYL<br>DQTPRVFYLA<br>DRPSIGNLAGANSLL<br>DTAEKLRSPDDERK<br>EQVRALAPTKK<br>ESFFFPFELPREER<br>ESFFFPFELPREERG<br>ESLFFPFELPSEERGR<br>ESYFVDAQPKKKEEGN<br>FADAQPLQKEEGKKG<br>FKAVQDTYENAK<br>FLEHAFSVDK<br>FLVPPQESQKR<br>FNSKPNTLLLPHHAD<br>FNTNEDIAEK<br>FSKHFLAQSFNTNE		

FTPLTVTPQGEYNVRVSS<br>GARQLEENL<br>GNWGPLVNPESQQGSPR<br>GPLVNPESQQGSPR<br>HAIATGDLVSL<br>HHTNPTKPINL<br>IAEKLQSPDDERK<br>IQHTFNLKSQQAR<br>IVEISKEQIR<br>IVEISKEQIRALSK<br>IVKEGQLASQYR<br>KAHFTAPSSVR<br>KEAGAKMELHEAKAR<br>KFLVPPQESQKR<br>KIKEKLPGGHSD<br>KIKEKLPGGHSDK<br>KPLVVQFQKLDKES<br>KQIVTVEGGL<br>KQTDEYGNPVHAA<br>KQTDEYGNPVHAASVTY<br>LAFPGSAK<br>LAFPGSAKDIENLIK<br>LAFPGSAQAVEKL<br>LAFPGSAQAVEKLLK<br>LAFPGSAQAVEKLLKN<br>LAFPGSAQAVEKLLKNQ<br>LAFPGSAQAVEKLLKNQRE<br>LAFPGSAQAVEKLLKNQRESY<br>LAFPGSAQDVERLLK<br>LAQSFNTNEDIAEK<br>LDTSNFNNQLDQTPRVF<br>LDTSNFNNQLDQTPRVFY<br>LEHAFSVDKQIAKNLQGENEGEDKGAIVTVKGGL<br>LEHAFVVDR<br>LKEAFGVNM<br>LKEAFGVNMQ<br>LLKNQRESYF<br>LNALPEEVIQHTFNLK<br>LQSPDDERKQIV<br>LQSPDDERKQIVTVEGGL<br>LRSPDDERKQIVTVEGGL<br>LVLPVQNDASTGLH<br>LVPPQESQKR<br>LVPPQESQR<br>LVPPQESQRRA<br>LVVGEVEVSK		

MDWIGAYRG<br>MLGGLLHR<br>MQIVRNLQGENEEEDSGAIVT<br>MQIVRNLQGENEEEDSGAIVTVKGGLR<br>NALEPDHRVESEGGL<br>NALPEEVIQHTFNLK<br>NALPEEVIQHTFNLKSQQAR<br>NVTVPKVEKKPENKNVR<br>PALSWLRL<br>PEEVIQHTF<br>PEEVIQHTFNLK<br>PEEVIQHTFNLKSQQAR<br>RADLPGVKKEDLK<br>RAIPSEVLAHSYNLRQSQ<br>RGHKIKGTVVL<br>RPSIGNLAGANSL<br>RPSIGNLAGANSLL<br>RPSIGNLAGANSLLNALPEE<br>RQFQLSAQ<br>SGAIVTVKGGLR<br>SGFSKHFLAQSFNTNE<br>SGIIHKIEETL<br>SKHFLAQSFNTNE<br>SKHFLAQSFNTNEDIAEK<br>SKHFLAQSFNTNEDTAEK<br>SPDDERKQIVTVEGGL<br>SQLLDKAKNFVSEK<br>SQVRQLKYQGNSGPLVNP<br>STIVGSSGGTMISTSTPHMVL<br>TAVGWAAAHFSAEK<br>TFNLKSQQAR<br>TKDYASDATDAAKK<br>TLEFLEHA<br>TNDRPSIGNLAGANSLL<br>TPMIGTLAGANSLL<br>VIQHTFNLK<br>VSELKYEGNWGPLVNPESQQGSPR<br>VVGEVEVSK<br>VVLPVQNDGSTGLHWANLQK<br>YVVNPDNNENLRL<br>AAHAIATGDL<br>AGANSLLNAL<br>AQQLPKQAQVK<br>DIAEKLQSPDDERKQIVTVEGGL<br>FAIGINAENNQRN<br>FNKRSQQLQNLRD		

IIIAQGKGAL<br>KLQSPDDERKQIVTVEGGL<br>LKYEGNWGPLVNPESQQGSPR<br>NQRSPQLQNL<br>QIKNNNPFK<br>QIVRNLQGEN<br>RVTAPAMRKPQQEEDDDDE<br>SRYPTSKGAIIFGDAPNN<br>SVIKPPTDEQQQRPQE<br>SVIKPPTDEQQQRPQEE<br>VRLVLPGEL<br>AIHATQGSTQQLGPLVTVPQFL<br>ALEPDHRVESEGGL<br>ERKQIVTVEGGL<br>FKNQYGHVR<br>FKNQYGHVRV<br>FNLKSQQAR<br>FVDAQPQQKEEGSKGR<br>FVDAQPQQKEEGSKGRKG<br>FVDAQPQQKEEGSKGRKGP<br>GALLLPHF<br>ITLAIPVNKPGRFESF<br>KGAIVTVKGGL<br>KLLKNQRESY<br>KTISSEDKPFNLR<br>KTNDRPSIGNLAGA<br>KTNDRPSIGNLAGAN<br>KTNDRPSIGNLAGANSL<br>KYEGNWGPLVNPESQQGSPR<br>LAFPGSAQAVEK<br>LAFPGSAQDVERL<br>LAIPVNKPGRFESF<br>LDTSNFNNQLDQTPR<br>LKYQGNSGPLVNP<br>LLKNQRESY<br>NALEPDHRVE<br>NKRSQQLQNLRD<br>NQRSPQLQNLRD<br>RAIPSEVL<br>RKQIVTVEGGL<br>VEKLLKNQRESY<br>VIQHTFNLKSQQAR<br>VIQHTFNLKSQQARQ<br>VPIPQHHTNPTKPINL<br>VRQLKYQGNSGPLVNP<br>VTPTKPINL		

YEGNWGPLVNPESQQGSPR		
